# Large Language Models’ Simulation of Patient Comprehension of Discharge Summaries: Multimodel Evaluation Study

**DOI:** 10.2196/89003

**Published:** 2026-10-22

**Authors:** Xinyao Ma, Rui Zhu, Zihao Wang, Jingwei Xiong, Qingyu Chen, L Jean Camp, Haixu Tang

**Affiliations:** 1 Luddy School of Informatics, Computing, and Engineering, Indiana University Bloomington, 700 N Woodlawn Ave, Bloomington, IN, United States, 1 812-223-3981; 2 School of Medicine, Yale University, New Haven, CT, United States; 3 College of Computing and Data Science, Nanyang Technological University, Singapore, Singapore; 4 Biostatistics, University of California, Davis, Davis, CA, United States; 5 College of Computing and Informatics, University of North Carolina at Charlotte, Charlotte, NC, United States

**Keywords:** artificial intelligence, AI, machine learning, digital health, health informatics, fairness, bias, and ethics in AI

## Abstract

**Background:**

Large language models (LLMs) are increasingly used to generate and evaluate patient-facing medical materials. Through persona prompting, LLMs can be instructed to simulate responses from individuals with different demographic and experiential backgrounds, offering a scalable approach for approximating patient feedback. However, it remains unclear how closely these simulated persona-based responses reflect real patient perspectives, and whether this alignment is consistent across demographic subgroups.

**Objective:**

This study assessed whether contemporary LLMs can simulate diverse patient perspectives on hospital discharge summaries when prompted with personas defined by education, gender, and health care use. We further examined whether persona-based simulation performance was equitable across subgroups by comparing LLM-generated responses with responses from a general-population human cohort under a standardized evaluation framework.

**Methods:**

We evaluated 4 contemporary LLMs, GPT-4.1, GPT-5.2, Claude Sonnet 4.5, and Opus 4.6, against responses from 99 participants recruited through Prolific (Prolific Academic Ltd). Participants answered 10 questions for discharge summaries that varied in length and medical complexity. Questions included information-based items assessing factual comprehension and perception-based items assessing subjective understanding. Model-human alignment was measured using modal mean absolute error and expected absolute ordinal distance. We also conducted a chain-of-thought sensitivity analysis for cases with the greatest divergence between LLM-generated persona and matched human responses.

**Results:**

Across LLMs, persona responses aligned more closely with human responses on information-based items than on perception-based items. Education was the most consistent stratification axis: every model aligned more closely with the high-education subgroup than with the low-education subgroup on information items, suggesting that LLMs capture broad factual-comprehension differences associated with educational background. Perception items, in which subjective understanding and uncertainty dominate human responses, were simulated less reliably. Alignment also depended on discharge summary characteristics, with short but clinically complex summaries producing the largest divergence. Gender-related differences were smaller and inconsistent in direction, reflecting item-level modal flips rather than systematic distributional bias. Chain-of-thought improved alignment on some factual items but did not recover the full range of less common human responses. Across models, LLMs underselected “I don’t know” and “Not provided” responses relative to humans, indicating a tendency to commit to determinate answers even when human participants expressed uncertainty.

**Conclusions:**

Contemporary LLMs can approximate common response patterns within demographic subgroups, particularly for factual comprehension and education-based personas. However, they are less reliable for capturing heterogeneous and perception-based patient responses. Equity performance varied substantially across models, making model selection an important design consideration. Persona-based LLM simulation should be used as an assistive screening tool for patient materials and should complement, not replace, human evaluation with subgroup validation.

## Introduction

### Background

Large language models (LLMs) have rapidly advanced natural language processing by demonstrating broad capabilities across text generation and complex reasoning tasks [1-3]. Central to their versatility is in-context learning (ICL), which enables the same pretrained model to adapt to new prompts, roles, and instructions without additional training [4]. By leveraging ICL, LLMs can be prompted to take on different perspectives, for example, to reason as individuals with varying demographic, professional, or experiential backgrounds. This role-play capability has been applied to tasks ranging from exploring demographic biases [5] to serving as subjective or objective evaluators in summarization and coding [6,7], and it is the starting point of our work.

Health care is one domain in which this adaptive capability has immediate utility, particularly for improving the clarity of patient-facing medical documents. Hospital discharge summaries carry essential information about diagnoses, medications, and follow-up care, yet are often difficult for patients and caregivers with limited medical training to fully understand [8]. LLMs have been applied to generate and assist with such documents to preserve clinical accuracy while improving readability [9,10], and early work suggests these tools can reduce documentation burden on clinicians [11,12]. Adoption, however, introduces risks: LLMs can inherit biases from their training data [13] that surface in generated outputs [14-16] and raise fairness concerns [17-20], and their articulate language may induce automation bias under which model responses are trusted beyond what the evidence warrants [21]. Patient-centric evaluation is therefore needed for clarity and safety as well as clinical accuracy, but gathering such feedback is slow and difficult to scale to every revision of every document.

One way in which LLMs extend beyond content generation is by repurposing the same adaptive capability to evaluate the documents they generate. A single model can be prompted to reason as a medical professional, checking technical accuracy, and, in a separate session, as a patient or family member with a limited medical background, asked whether the document is clear, the tone reassuring, and specific clinical information locatable. This adaptability makes LLMs a candidate scalable tool for pretesting patient-facing communications across audiences with very different needs. The technical mechanism is ICL [4,22-27]: a persona-based prompt such as “act as an elderly patient with low health literacy” [28-31] steers the model toward the feedback a real person in that situation might provide [32,33], offering a pathway to pretesting patient-facing communications at a fraction of the cost of a human study.

The critical question that remains underexamined is how faithfully persona-conditioned LLMs reproduce the responses of real patients from the simulated demographic group, and whether that fidelity is equitable across subgroups. Prior work on LLMs in health care has predominantly studied clinical text generation [34], expert task performance [35], virtual patient education [36], and provider-facing applications [37]; the use of LLMs as demographic-sensitive proxies for general-population patient perspectives on discharge summary clarity remains understudied. This gap matters because an LLM that faithfully simulates highly educated or male patients but poorly simulates lower-education or female patients would systematically underrepresent exactly the populations most likely to benefit from improved discharge communication, silently entrenching existing health-literacy gaps.

In this study, we evaluate whether, and under what conditions, LLMs can approximate the response patterns of a diverse human cohort answering questions about hospital discharge summaries. We benchmark 4 mainstream models: GPT-4.1, GPT-5.2, Claude Sonnet 4.5, and Opus 4.6, against responses from 99 general-population participants. The evaluation uses 4 discharge summaries from MIMIC-IV (Medical Information Mart for Intensive Care) selected to vary along 2 document-level axes: length and medical complexity; and a 10-item questionnaire containing both factual-recall, information-based questions and subjective, perception-based questions.

Using a standardized decoding protocol applied as consistently as the provider APIs allowed, temperature 1.0, 10 iterations per persona-question cell, default penalty settings, we condition each model on personas stratified by education, gender, frequency of doctor visits, and frequency of emergency department visits. We then quantify alignment between each persona-conditioned model distribution and the corresponding matched human subgroup using modal mean absolute error (MAE), expected absolute ordinal distance, and a human split-half baseline as an empirical reference for within-subgroup variability. Finally, we conduct a targeted chain-of-thought (CoT) sensitivity analysis to test whether alignment gaps persist when models are prompted to generate an explicit rationale before selecting an answer.

### Study Objectives

#### Overview

The aim of this study is to characterize both the capability and the equity of persona-conditioned LLM simulation of patient perspectives on discharge summaries, and to identify the conditions under which such simulation is, or is not, a reliable substitute for human evaluation. Based on the preceding literature, we test 3 prespecified hypotheses.

#### H1 (Task-Type)

Persona-conditioned LLMs will align with human responses more closely on information-based (factual-recall) questions than on perception-based (subjective) questions.

#### H2 (Demographic-Equity)

Persona-based alignment will be most reliable when the persona is stratified by education level, and less reliable when stratified by gender, doctor visits, or emergency department visits. Alignment gaps between paired subgroups (eg, low-education vs high-education) will exceed the human split-half noise floor.

#### H3 (Document-Dependence)

The magnitude and direction of education-related alignment gaps will depend on the length and medical complexity of the discharge summary, rather than being constant across documents.

We return to these hypotheses in the “Discussion” section, where we also consider the implications of the observed patterns for the safe deployment of LLMs in patient-centric communication.

## Methods

### Ethical Considerations

The study followed the ethical standards for human participant research. All participant recruitment and online survey data collection procedures were reviewed and approved by the Indiana University Institutional Review Board (IRB; protocol number 23323). All data analysis was subject to IRB review and approval.

Participants were presented with a study information sheet prior to engagement, which detailed the research objectives, the voluntary nature of the study, and their right to withdraw at any time without penalty. Participants were told that those who completed the survey would be compensated with US $3 distributed through the Prolific platform (Prolific Academic Ltd). During informed consent, participants were told compensation would be increased by US $1 for accurate answers. We set an attention-check question: “Select 4 if you see this question: A. 1 B. 2 C. 3 D. 4.” We consider participants who correctly selected as “accurate.” Finally, 99 participants completed the study, correctly selected the attention question, and received US $4. Electronic informed consent was obtained from all participants before they proceeded to the survey.

To ensure privacy and confidentiality, all participant data were anonymized by the Prolific platform before being accessed by researchers. Access to these data was strictly limited to researchers listed on the approved IRB protocol. No personally identifiable information was solicited or stored in conjunction with the research findings. Furthermore, no images or identifiable data of individual participants are included in the dataset, the paper, or its supplementary materials.

### Study Design

To investigate how well an LLM can simulate patient perspectives, we conducted a 2-arm comparison in which the same 4 discharge summaries and the same 10-item questionnaire were given to (1) human participants recruited from a general-population online panel and (2) persona-conditioned LLMs prompted to adopt demographic identities matched to the human subgroups. Figure 1 summarizes the overall experimental workflow. After reading an informed consent, human participants completed an online survey preceded by the instruction, “Imagine that you are a patient who has received medication instructions from your physician. Please read the following medical discharge summary and answer the questions that follow.” For the language model, the same discharge summaries and questions were presented to each LLM under a demographic persona prompt. Details of the human survey and demographic composition are described in the “Human Evaluation” section below; details of the persona prompt, model roster, and decoding protocol are described in the “In-context Impersonation of LLMs” section.

**Figure 1.**
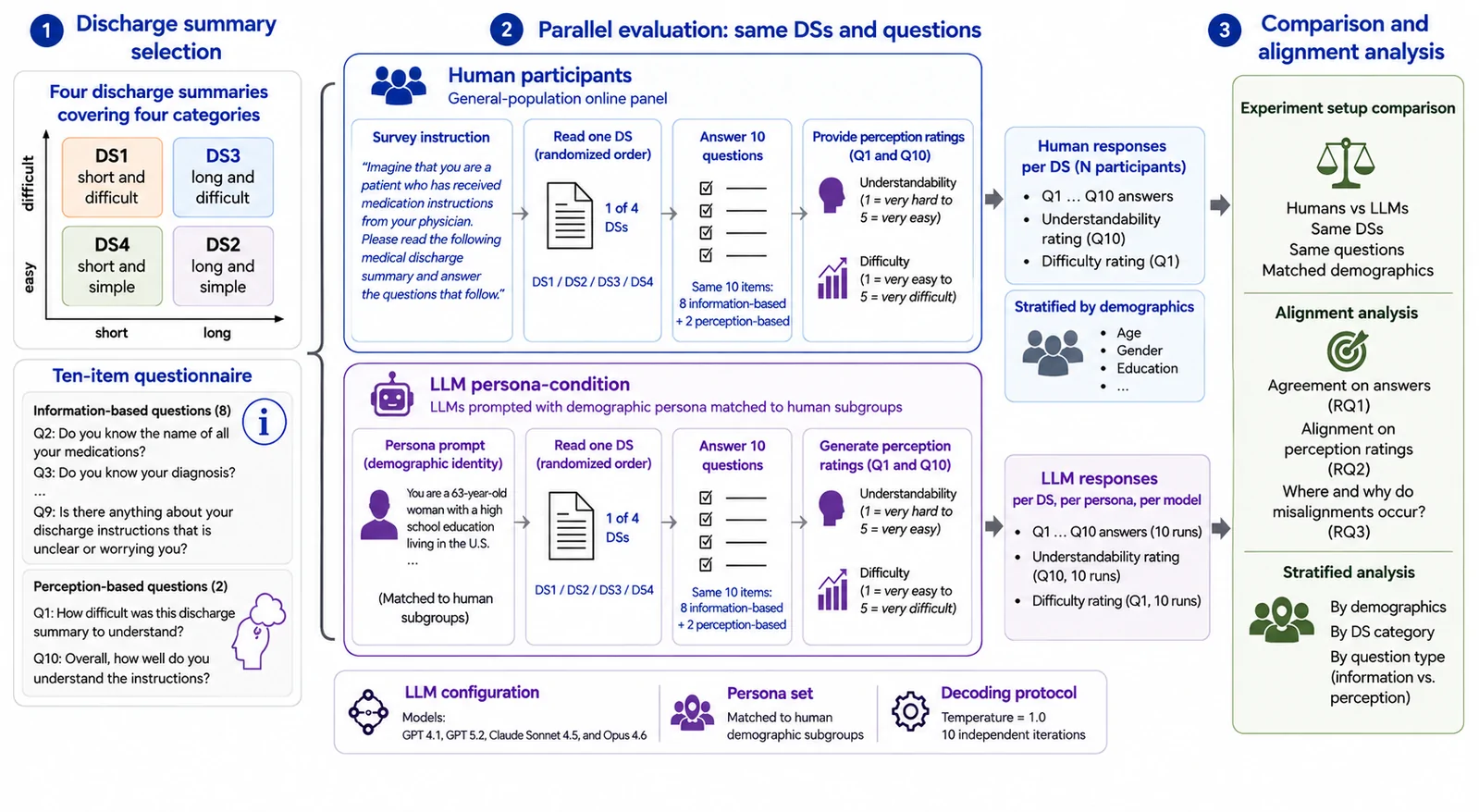
Overview of the study design. DS: discharge summary; LLM: large language model.

The 4 discharge summaries (denoted as DS1-DS4) used in this study were sourced from a large, anonymized medical database—MIMIC-IV [38], with a specific focus on sections intended for direct patient communication. We selected the summaries based on 2 key factors: length (short vs long) and complexity (simple vs difficult), which was determined by the density of professional medical terminology. This resulted in 4 summaries, with one summary chosen to represent each: DS1: short and difficult, DS2: long and simple, DS3: long and difficult, and DS4: short and simple. Each selected summary contained details on medications, follow-up treatments, and care recommendations. Discharge summary complexity was defined using qualitative criteria (density of medical abbreviations, jargon, and sentence length).

### In-Context Impersonation of LLMs

LLMs are trained primarily through next-token prediction, learning to estimate the probability of a token conditional on the preceding context. This objective enables models to acquire linguistic patterns and generate coherent natural-language responses. In this study, we used off-the-shelf API accessible LLMs without any task-specific fine-tuning, relying on their existing language-generation capabilities. Specifically, we evaluated 4 mainstream LLMs: GPT-4.1 and GPT-5.2, accessed through the OpenAI API, and Claude Sonnet 4.5 and Opus 4.6, accessed through the Anthropic API. The dated model snapshots used during data collection were claude-sonnet-4-5-20241022 (Sonnet 4.5) and claude-opus-4‐6 (Opus 4.6), both via the Anthropic API; and gpt-4.1-2025-04-14 (GPT-4.1) and gpt-5.2-2025-12-11 (GPT-5.2), both via the OpenAI API.

To promote methodological consistency across models, we standardized decoding settings as closely as the provider APIs allowed. The sampling temperature was fixed at 1.0 to allow stochastic response variation across repeated queries. Nucleus sampling was otherwise left unconstrained, with top p kept at its default value of 1.0 where supported. For OpenAI models, frequency penalty and presence penalty were kept at their default values of 0.0; the Anthropic API does not expose directly equivalent penalty parameters. No stop sequences, logit biases, or additional system or developer instructions beyond the persona prompt described below were applied.

Under these settings, each question was queried in 10 independent iterations per persona per model. This repeated-sampling design produced empirical synthetic response distributions rather than single-response point estimates, enabling direct comparison between model-generated response distributions and the observed response distributions of matched human subgroups.

For each task, we constructed a task-specific context that described the discharge summary scenario, presented the multiple-choice question, and specified the required response format. To evaluate persona-conditioned responding, we prefixed this context with an impersonation prompt of the form “You are a {age} {gender} with {education} level...,” where persona denoted the demographic or experiential subgroup being simulated. The persona prompt conditioned the model on 6 attributes. Four attributes were stratification axes for the equity-gap analyses: education (low or high), gender (male or female), doctor-visit frequency (low or high), and emergency room (ER)–visit frequency (low or high). Two were varied in the prompt but not analyzed separately: age (25, 45, and 65 years; anchoring early adulthood, middle age, and the older adult population most likely to receive discharge instructions) and self-reported ethnicity (White, Black, Hispanic, and Asian; the most common US patient-population categories). We refer to this prompting strategy as in-context impersonation: the model is asked to condition its response on a stated identity using only information provided in the prompt, without any task-specific retraining or fine-tuning.

Operationally, the LLM generates a response token by token, with each token sampled conditional on the full preceding context, including the task instructions, the assigned persona, and any tokens already generated. Thus, the persona prompt enters the same context window as the discharge summary and question, allowing the model to condition its response on the assigned subgroup description through ICL. We do not assume that the model literally adopts the perspective of the specified group; rather, we use this design to test whether persona-conditioned response distributions resemble the response distributions observed among matched human participants [8,39,40].

To evaluate the LLMs’ ability to approximate patient-perspective response patterns, we tested persona conditions across 4 variables: education level, gender, frequency of doctor visits, and frequency of ER visits. The persona prompts instructed the model to answer from a specified subgroup identity, using formulations such as “you are a 25, male with low education level.” After receiving the persona prompt, the model was shown a discharge summary and asked to answer questions from that assigned perspective. The response instructions required the model to output only the selected answer option, such as A, B, C, or D, without additional explanation. A complete example prompt is provided in Multimedia Appendix 1.

### Human Evaluation

To assess the comprehensibility of the 4 discharge summaries from a human perspective, we conducted an online survey of 100 general-population participants, of whom 99 produced usable data (one was excluded for incomplete responses). Each participant was evenly and randomly assigned to read 3 of the 4 discharge summaries and, after reading each one, answered 10 items. The 10 items were divided into 2 categories: information-based (8 questions on factual-recall) and perception-based (2 questions assessing subjective understanding and the participant’s rating of the summary’s clarity and difficulty). Each participant also completed 6 demographic questions once, yielding 36 total responses per participant (3 summaries × 10 items +6 demographics).

The 8 information-based questions were designed to test whether participants could accurately extract key facts from the discharge summary. These questions included direct recall prompts such as “Do you know the name of all your medications?” and “Do you know your diagnosis?” as well as questions requiring deeper comprehension, such as “Do you know what kind of treatment you need to follow based on the discharge instructions?” The goal was to objectively measure each participant’s ability to acquire and understand the provided medical information.

In contrast, the 2 perception-based questions assessed the participant’s subjective feeling of comprehension. Immediately after reading the summary, the first question asked participants to rate their understanding on a scale from “Very clear” to “Not clear at all.” To cross-validate this self-assessment, a second question was placed at the end of the survey, asking participants to rate the summary’s difficulty on a scale from “Extremely easy” to “Extremely difficult.” A complete list of the questions and summaries used in the experiment is available in Multimedia Appendix 1.

### Human Responses

We recruited participants through the Prolific platform, initially grouping them based on their highest educational degree on record. Each participant was presented with 3 randomized discharge summaries to mitigate order effects and reduce fatigue. For each summary, they were required to answer follow-up questions and were permitted to refer to the text as needed. In addition to the demographic data provided by Prolific, our survey included questions about self-reported education, race, and frequency of doctor and ER visits. A full list of these questions is available in Multimedia Appendix 1.

We collected 99 valid responses from a cohort with diverse educational backgrounds. Of these, 50 participants reported holding a college or postgraduate degree (high-education subgroup), 48 participants reported educational attainment below a college degree (low-education subgroup), and 1 participant did not report their highest educational degree and was therefore excluded from education-stratified analyses. A more detailed demographic breakdown is provided in Table 1.

**Table 1.** Demographics of human survey participants (N=99).

Item and category	Participants, n
Sex	
Female	58
Male	35
Nonbinary and third gender	6
Age (years)	
18‐24	21
25‐34	38
35‐44	23
45‐54	11
55‐64	5
≥65	1
Ethnicity^a^	
White and Caucasian	65
Black and African American	15
Hispanic and Latino	5
Asian	3
Native American	1
Multiracial	10
Education^b^	
No post-high school diploma	3
Some high school	5
High school graduate	28
Some college	12
College graduate	39
Postgraduate degree	11
Doctor visit frequency	
Weekly	2
Monthly	21
Yearly	72
Never	4
ER^c^ visit frequency	
Weekly	1
Monthly	2
Yearly	51
Never	45

a Multiracial: respondents selecting more than one ethnicity (n=10); Black and African American includes one “Black American” response (n=15 total).

b One respondent skipped the education question (nedu=98). For binary analyses: low=no diploma + some high school+ high school graduate + some college (n=48); high=college graduate + postgraduate (n=50).

c ER: emergency room.

### Statistical Analysis

All response-level comparisons between LLM and human subgroups were based on the full synthetic response distributions generated by the 10-iteration sampling procedure. For each matched subgroup pair, we report modal MAE and expected absolute distance (EAD) as the primary alignment metrics. We additionally report Jensen-Shannon divergence (JSD, base 2) in the CoT sensitivity analysis.

Although the survey reuses Q-numbers across discharge summaries, 3 items contain DS-specific content (Q6 on DS2, and Q7 on DS2 and DS3), and each item is scored against the version actually presented to the respondent. Items Q6 to Q9 are check-all-that-apply questions; for these, each chosen option contributes one vote to the response distribution. Items Q2 through Q5 form a confidence and certainty axis (“Yes,” “I don’t know”and “Not provided”) and admit an ordinal interpretation; items Q6 through Q9 are categorical. We report modal MAE and EAD on the full information-question block and add a complementary exact-match alignment rate so that conclusions can be assessed under either the ordinal or the exact-match metric.

To contextualize LLM-human alignment against natural within-subgroup variability, we computed a human split-half baseline. Within each human subgroup, respondents were randomly partitioned into two halves, and the same alignment metric was computed between the halves. This procedure was repeated over 200 random split-half resamples. The resulting baseline estimates the alignment distance expected from finite sample size and within-subgroup disagreement. LLM-human distances that substantially exceeded the split-half baseline were therefore interpreted as evidence of divergence beyond ordinary within-subgroup variability, rather than as differences attributable solely to sampling noise.

### Sensitivity Analysis: CoT Influence

To assess whether the suppression of explicit reasoning traces affected simulation fidelity, we conducted a targeted sensitivity analysis on a representative subset of our data. We selected Sonnet 4.5 and GPT-5.2 for reevaluation on DS1 (information-based questions) and DS3 (perception-based questions). In the sensitivity analysis, models were additionally prompted to generate a brief reasoning trace prior to option selection. Initial results suggested that although CoT prompting slightly increased response diversity on perception tasks, underlying positional biases (eg, Sonnet’s preference for option B) persisted, indicating that the observed alignment gaps likely arise from the internal parameters of the model rather than from prompt-level suppression of reasoning.

The two prompting conditions differed only in the instruction appended before the discharge summary. In the zero-shot condition, the model was instructed to “Answer with only the letter.” In the CoT condition, the model was instructed to “First, explain how this person would interpret the summary and why they would choose a specific option. Then, on a new line, provide the letter of your answer.” All queries used a single default persona to isolate the effect of the prompting strategy and remove demographic conditioning as a confound. Each question was queried 13 times per condition, with the temperature set at 1.0. For CoT responses, the selected letter was extracted as the last capital letter on its own line, with a fallback to the first (A-G) token encountered. Alignment with human responses was quantified using the JSD (base 2, lower values indicate closer alignment with humans) between the model’s empirical distribution over the applicable option set and the human reference distribution:

$JSD(P∥Q)=\frac{1}{2}D_{KL}(P∥M)+\frac{1}{2}D_{KL}(Q∥M),\;M=\frac{1}{2}(P+Q)$

.

In scenario A (DS1, Q2-Q9, and information-based), Claude Sonnet 4.5 overselected option B (54.8%) relative to the human modal preference for option A (52.9%). In scenario B (DS3, Q1, and Q10, and perception-based), the human distribution exhibited the greatest diversity (modal B=49.7%). See the “CoT Versus Zero-Shot Prompting” section for detailed analysis and results.

## Results

### Overview

We compared persona-conditioned LLM responses with responses from 99 general-population participants across all evaluation items. Taken together, the results show that LLMs can approximate some aggregate patient-perspective response patterns, particularly when responses are stratified by educational background, but that simulation fidelity varies substantially by question type, subgroup definition, and discharge summary characteristics. In the following sections, we characterize the distributional properties of the human responses and quantify alignment between each persona-conditioned LLM subgroup and the corresponding human subgroup. Six key findings emerge from this analysis; each is highlighted as a shaded callout at the end of the corresponding section.

### Distribution of Human Responses by Gender and Education

Before evaluating LLM persona simulation, we first characterized the human response patterns. Aggregate response distributions were nearly identical across self-reported gender groups: male, female, nonbinary; normalized Shannon entropy >0.93, and across education levels: low versus high: 21% versus 21% (A), 27% versus 26% (B), 32% versus 32% (C), and 14% versus 14% (D); normalized Shannon entropy >0.94. (Table S1 in Multimedia Appendix 1). We only consider the A-D answer distribution because E, F, or G are excluded from the pooled statistic (1.5%‐2.6% of responses per subgroup). The high $H_{n}^{\left(A\text{-}D\right)}\approx 0.95$and ${\bar{H}}_{n}(per\text{-}Q)\approx 0.48$ for male and female, low and high education groups indicate distributed responses relatively evenly across A-D, and at the individual-question level, responses become somewhat more concentrated. These results indicate substantial within-group response variability and no systematic aggregate preference along either demographic axis. Because aggregate distributions can obscure consistent item-level patterns, we next examined within-group agreement at the question level.

Within-group pairwise agreement was 51%‐55% overall. Agreement was driven primarily by information-based items, where pairwise agreement reached 54% among male participants and 60% among female participants, but was substantially lower on perception-based items, at 39% and 37%, respectively. Despite this variability, male and female participants agree on the plurality response for 35 of 40 (87.5%) questions, while low- and high-education participants agree on 33 of 40 (82.5%), and perception item agreement was nearly identical across education subgroups, at 37%‐38% (Table S2 in Multimedia Appendix 1). All within-group agreement values exceed the 25% random baseline by more than 2× on information items and by more than 1.5× on perception items, confirming meaningful within-subgroup consensus that the LLMs fail to reproduce.

Thus, response variability on perception questions appears to reflect actual subjective heterogeneity rather than systematic demographic divergence. When humans within a gender or education subgroup disagreed on a perception question, those disagreements did not consistently align with demographic boundaries. This imposes an important constraint on the LLM analysis that follows: a persona-conditioned model that produces a narrow, mode-concentrated response distribution may appear well aligned on information-based items, where human responses show relatively high within-group agreement, but will be structurally unable to reproduce the broader dispersion observed on perception-based items.

### Robustness Check: High-School-Only Versus Merged Low-Education Reference

The low-education group (n=48) combined 3 educational sublevels: some college (n=12), high school graduate (n=28), and some high school or less (n=8; combining the “Some high school” category [n=5] and the “No post-high-school diploma” category [n=3] in Table 1). To assess whether respondents with some college experience attenuated the low-education signal, we repeated the education-stratified analysis using a stricter high-school-or-less reference group (n=36), excluding the 12 respondents with some college experience.

This sensitivity analysis produced minimal changes. Alignment scores shifted by at most ±0.031, corresponding to 1 question out of 32 (Table S3 in Multimedia Appendix 1). Sonnet 4.5, Opus 4.6, and GPT-5.2 each gained one additional information-item alignment match under the stricter high-school-or-less reference, whereas GPT-4.1 lost one match: it aligned with option C on DS3-Q5 under the merged low-education definition, but the reference response changed to option A when the some-college respondents were excluded. Thus, the original low-education grouping is robust. Excluding respondents with some college experience does not strengthen the observed education effect, change any alignment rankings, or alter the substantive model comparisons reported in the main text.

### Alignment Between LLM and Human Responses

After characterizing the human response distributions, we evaluated how closely each LLM reproduced those distributions when conditioned on the corresponding demographic persona. Figure 2 shows the density and variance of human and LLM-generated responses overall, and Figure 3 is stratified by question type. Tables 2 and 3 report distance-based alignment between each LLM-generated subgroup and the matched human subgroup. For example, responses generated under the low-education persona condition were compared with responses from participants in the low-education human subgroup.

**Figure 2.**
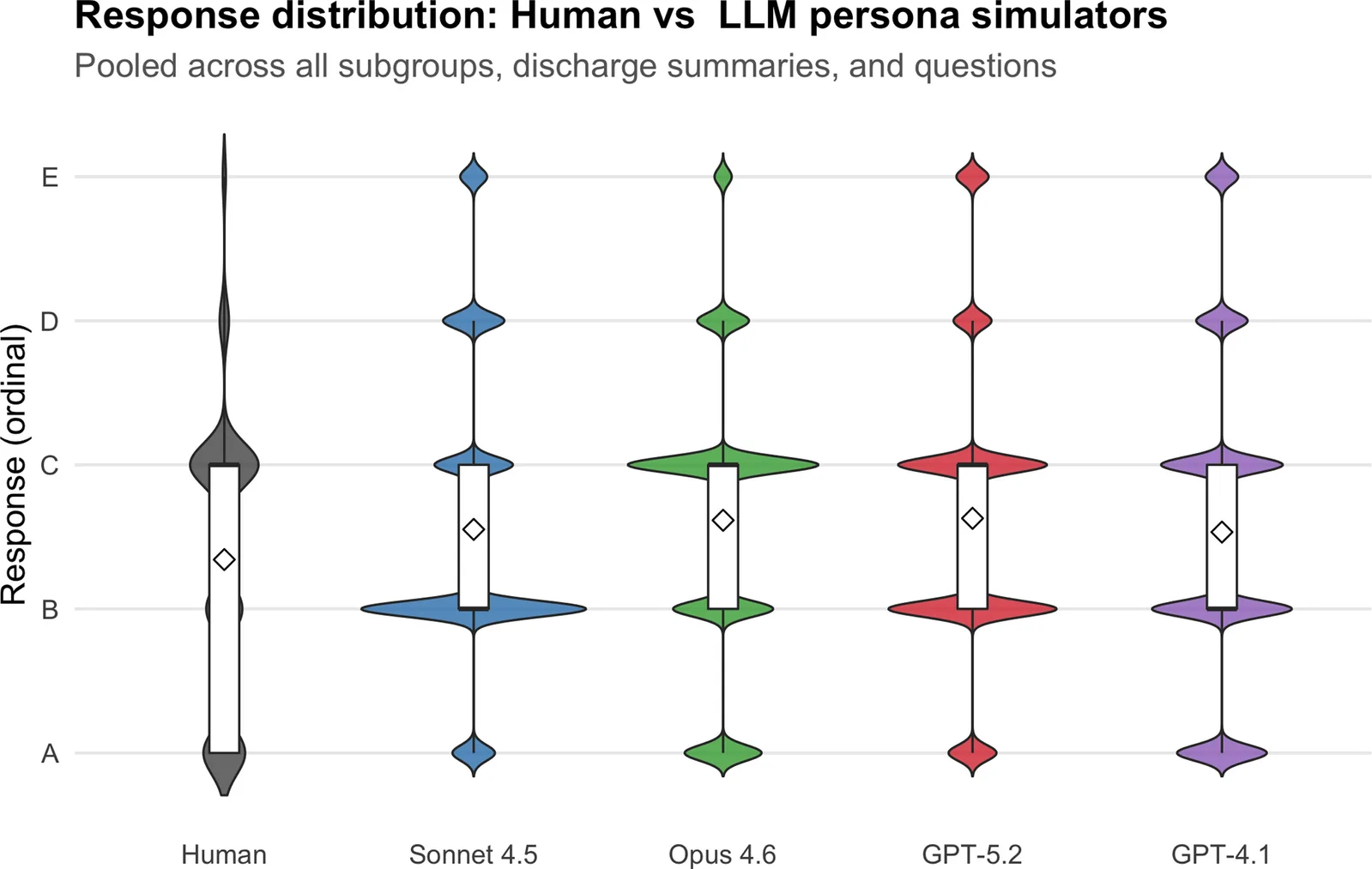
Density and variance of human and model responses: overall distribution. LLM: large language model.

**Figure 3.**
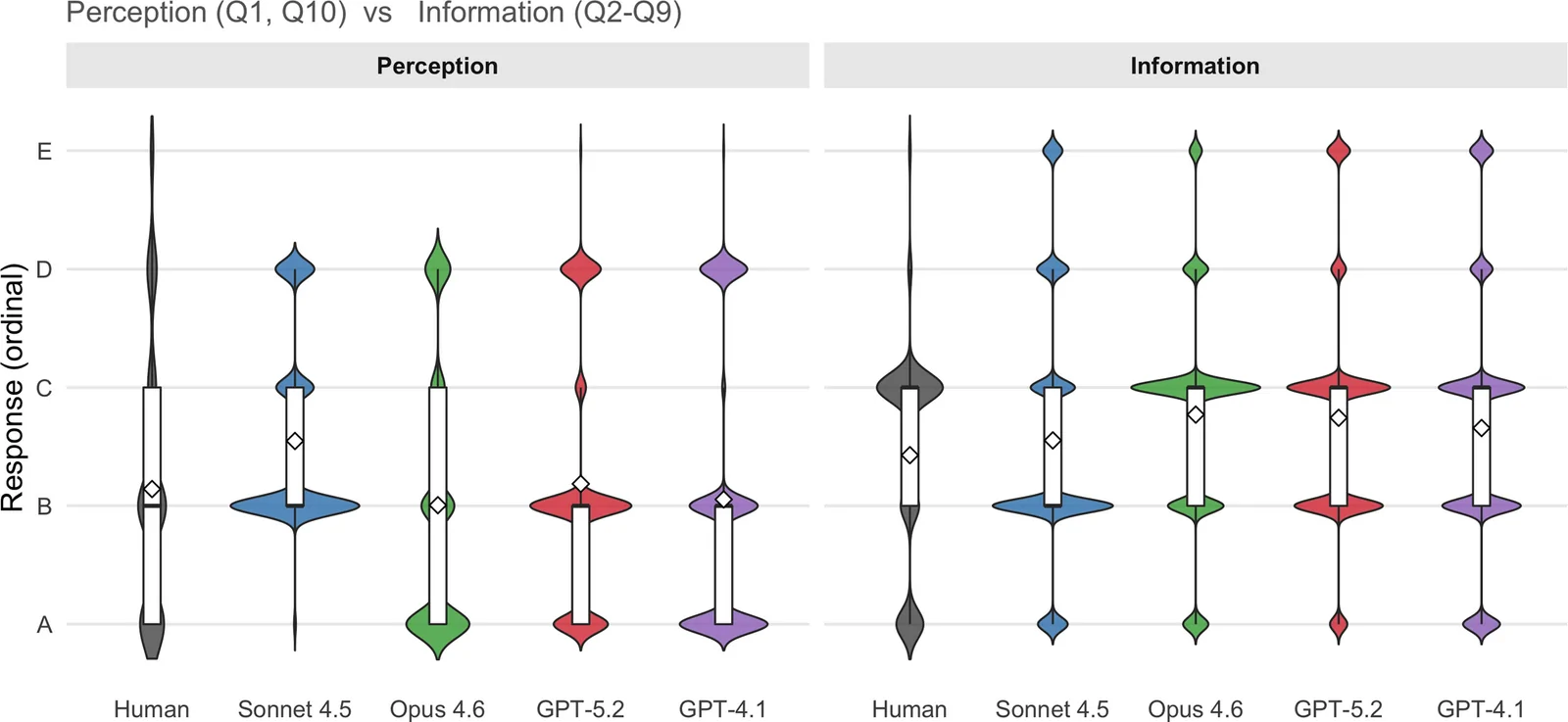
Density and variance of human and model responses for perception-based and information-based questions.

**Table 2.** Distance-based alignment between each large language model subgroup and the matched human subgroup, measured as modal mean absolute error^a^.

Model^b^	Education		Sex			Doctor visits		ER^c^ visits
	Low	High	Male	Female		Low	High	Low
Perception-based questions (Q1, Q10; n=8)								
Sonnet 4.5	1.63	0.75	0.75	0.63	0.75		0.88	0.63
Opus 4.6	1.38	1.25	1.13	1.13	1.00		0.38	1.00
GPT-5.2	1.13	1.25	0.88	0.50	0.75		1.00	0.88
GPT-4.1	1.38	1.13	1.25	0.63	0.88		1.13	1.13
Human (split-half)	0.38	0.65	0.27	0.59	0.60		0.80	0.37
Information-based question (Q2-Q9; n=32)								
Sonnet 4.5	0.91	0.88	0.88	0.91	0.94		0.97	0.88
Opus 4.6	0.66	0.63	0.78	0.66	0.78		0.72	0.66
GPT-5.2	0.78	0.59	0.72	0.78	0.81		0.75	0.69
GPT-4.1	0.91	0.78	0.75	0.75	0.75		0.75	0.75
Human (split-half)	0.44	0.36	0.52	0.26	0.34		0.49	0.32

a The upper block reports perception-based questions (Q1 and Q10 per discharge summary; n=8) and the lower block reports information-based questions (Q2-Q9 per discharge summary; n=32). Q2-Q5 are the confidence-axis items and Q6-Q9 are the categorical items. Lower values indicate closer alignment; the per-item maximum attainable distance varies from 2 (3-option items) to 5 (6-option items) across the 32 information items, with an item-weighted maximum of approximately 2.9; the 8 perception items have a uniform per-item maximum of 4. An exact-match alignment for information items is provided in Table S10 in Multimedia Appendix 1 and a per-item normalized version is provided in Table S11 in Multimedia Appendix 1.

b Values are rounded to 2 decimal places using round-half-up; equity gaps are computed from unrounded subgroup values and may therefore differ from the implied difference of the rounded subgroup values by at most 0.005.

c ER: emergency room.

**Table 3.** Distribution-level alignment between each LLM^a^ subgroup and the matched human subgroup, measured as EAD^b^, E [|ord (*Y*_LLM_) − ord (*Y*_Human_)|]^c^.

Model	Education		Sex		Doctor visits		ER^d^ visits
	Low	High	Male	Female	Low	High	Low
Perception-based questions (Q1, Q10; n=8)							
Sonnet 4.5	1.29	0.85	0.97	1.02	0.97	1.07	0.97
Opus 4.6	1.11	1.24	1.04	1.06	1.05	1.03	1.04
GPT-5.2	1.03	1.09	0.93	0.99	0.96	0.93	0.96
GPT-4.1	1.11	1.19	1	1.03	1.02	1	1.02
Human (split-half)	0.93	0.93	0.85	0.98	0.94	0.90	0.96
Information-based question (Q2-Q9; n=32)							
Sonnet 4.5	0.91	0.93	0.92	0.94	0.93	0.92	0.93
Opus 4.6	0.76	0.82	0.81	0.78	0.80	0.78	0.79
GPT-5.2	0.87	0.82	0.85	0.84	0.85	0.84	0.84
GPT-4.1	0.93	0.89	0.92	0.91	0.91	0.93	0.93
Human (split-half)	0.82	0.75	0.85	0.66	0.80	0.76	0.79

a LLM: large language model.

b EAD: expected absolute ordinal distance.

c The upper block reports perception-based questions (Q1 and Q10 per discharge summary; n=8) and the lower block reports information-based questions (Q2-Q9 per discharge summary; n=32). Q2-Q5 are the confidence-axis items and Q6-Q9 are the categorical items. Lower values indicate closer alignment; the per-item maximum attainable distance varies from 2 (3-option items) to 5 (6-option items) across the 32 information items, with an item-weighted maximum of approximately 2.9; the 8 perception items have a uniform per-item maximum of 4. An exact-match alignment for information items is provided in Table S10 in Multimedia Appendix 1 and a per-item normalized version is provided in Table S11 in Multimedia Appendix 1.

d ER: emergency room.

We report 2 complementary alignment metrics. Modal MAE (Table 2) measures the average ordinal distance, across questions, between the most frequently selected response option in the LLM-generated subgroup and the most frequently selected option in the corresponding human subgroup. EAD (Table 3) integrates over the full marginal response distributions, capturing distributional alignment beyond the modal response. For both metrics, lower values indicate closer alignment.

Across both metrics, LLM-human alignment was consistently stronger for information-based questions than for perception-based questions. For information-based items, all 4 models produced modal MAE values that were lower than those observed for perception-based items and closer to the human split-half baseline, although none reached within-human variability. For example, in the high-education subgroup, model modal MAE ranged from 0.65 to 1.06, compared with a split-half baseline of 0.32. These results suggest that persona-conditioned LLMs can often reproduce the dominant factual-recall response of a demographic subgroup, but less consistently than independent human samples from the same subgroup.

Perception-based questions showed a markedly different pattern. Modal MAE values were approximately 2- to 4-fold higher than the corresponding split-half baseline in nearly every condition. For example, in the low-education subgroup, one model produced a modal MAE of 1.62 compared with a split-half baseline of 0.39; in the high-education subgroup, another model produced a modal MAE of 1.25 compared with a baseline of 0.64. Thus, no evaluated LLM reproduced human perception-item response patterns at within-human fidelity. The violin plots in Figures 2 and 3 make this divergence visually apparent: human and LLM-generated distributions overlapped substantially on information-based items, whereas perception-based responses from LLMs were narrower and concentrated on 1 or 2 adjacent response options.

For information-based questions, only one pattern was consistent across all 4 models: every model aligned more closely with the high-education subgroup than with the low-education subgroup. The modal MAE advantage for edu-high ranged from 0.03 to 0.19 across models (mean 0.095, SD 0.079). Sex-related and outpatient use–related differences were smaller (|Δ|≤0.13 under modal MAE) and inconsistent in direction across models, indicating no systematic preference along these axes on factual-comprehension items.

Perception-based questions showed larger and less stable subgroup differences. The single largest education-related modal-MAE gap was +0.88 (Sonnet 4.5), and the largest sex-related gap was +0.63 (GPT-4.1, male worse), both substantially exceeding the corresponding information-block gaps. Outpatient-use gaps ranged from –0.25 to +0.63 across models, with no consistent direction. Because only 3 respondents reported high-frequency emergency department use (Table 1), the high-ED-visit subgroup was too small for stable alignment estimation, and we report values only for the low-ED-visit subgroup; for that subgroup, all 4 models produced alignment distances comparable to those observed for the low-outpatient-use subgroup.

Overall, LLM-human alignment was much closer to the human split-half baseline for information-based questions than for perception-based questions. This suggests that factual comprehension and subjective perception represent distinct challenges for persona-based simulation. An LLM may reproduce the most common factual response from a demographic subgroup reasonably well, while still failing to capture the broader variability in how real patients perceive the clarity or difficulty of the same discharge summary.

Finding 1 (Task-type performance): Across all 4 evaluated models, alignment with human responses is consistently closer for information-based questions than for perception-based questions. Modal MAE and EAD values are lower on information items in nearly every subgroup (Tables 1 and 3), indicating that factual-recall question types are more amenable to LLM persona simulation of general-population responses than are subjective perception items.

### Equity Gaps in Education and Gender

To quantify how equitably each model simulated different patient subpopulations, we computed the paired-subgroup gap (low-high for education; male-female for gender) on each of the 4 metric-by-question-type combinations; positive values indicate poorer alignment for the low education or male subgroup (Table 4).

**Table 4.** Education and gender equity gaps across all 4 models^a^.

Model	Modal MAE^b^		EAD^c^	EAD
EAD	Perception	Information	Perception	Information
Education gap (low-high)				
Sonnet 4.5	*0.88*	0.03	*0.44*	−0.02
Opus 4.6	0.13	0.03	−0.13	*−0.06*
GPT-5.2	−0.13	*0.19*	−0.06	0.04
GPT-4.1	0.25	0.13	−0.08	0.05
Gender gap (male-female)				
Sonnet 4.5	0.13	−0.03	−0.05	−0.02
Opus 4.6	0	*0.13*	−0.02	0.04
GPT-5.2	0.38	−0.06	*−0.06*	0.01
GPT-4.1	*0.63*	0	−0.03	0

a The upper block reports the education gap (edu-low minus edu-high); the lower block reports the gender gap. Positive values indicate poorer alignment for the low-education or male subgroup, respectively. Italicized values mark the largest absolute gap per metric column within each block.

b MAE: mean absolute error.

c EAD: expected absolute distance.

Sonnet 4.5 produced the largest education gap on perception items (modal MAE +0.88; 1.63 for edu-low vs 0.75 for edu-high, the largest disparity across all model-metric combinations; EAD +0.44), indicating that the model defaulted toward the response profile of highly educated respondents and failed to reproduce how lower-health-literacy patients perceived the summaries. Opus 4.6 was the most equitable on perception (modal MAE +0.13; EAD −0.13, slightly favoring edu-low). GPT-5.2 was the only model with better modal alignment for low-education personas on perception (gap=−0.13), but produced the largest education gap on information items (+0.19).

Gender gaps were smaller overall. GPT-4.1 showed the largest perception modal MAE gap (+0.63; male 1.25 vs female 0.63), but the corresponding EAD gap was only −0.03, indicating question-level modal flips rather than broad distributional mismatch. Opus 4.6 had the largest information-block gender gap (+0.13) and remained the most gender-equitable on perception (modal MAE gap=0.00); Sonnet 4.5’s information-question gender gap was effectively zero (−0.03). Across all models, EAD gender gaps were uniformly small (|Δ|≤0.06), confirming that gender-related differences largely reflect mode-switching sensitivity rather than systematic distributional bias.

Finding 2 (Demographic attribute impact): Persona-based alignment is most reliable when the persona is stratified by education level, whereas demographic attributes associated with greater behavioral variability, gender, and, by extension, health care use frequency (doctor and ER visits) yield lower and less stable alignment (Tables 2 and 4). Education is therefore the persona attribute most amenable to persona-based LLM simulation of general-population responses.

### CoT Versus Zero-Shot Prompting

#### Overview

We next conducted a sensitivity analysis comparing CoT and zero-shot prompting on the 2 scenarios with the largest model-human divergence in the main experiment: information-based questions on DS1 (scenario A) and perception-based questions on DS3 (scenario B). The impact of CoT was highly dependent on both model and task.

#### Scenario A: Information-Based Questions on DS1

In scenario A (Table S4 in Multimedia Appendix 1), CoT produced a substantial 66% reduction in JSD for Sonnet 4.5 (from 0.0847 to 0.0289), yielding the closest alignment to the human reference observed anywhere in this study, but a negligible change for GPT-5.2 (<1%), consistent with a near-deterministic response pattern on factual-recall items that was not altered by explicit reasoning. Both models nonetheless overrepresented option C (19%‐27% vs 6.8% for humans), suggesting consistent difficulty in distinguishing “Not provided” from “I don’t know” on factual-recall items.

#### Scenario B: Perception-Based Questions on DS3

In scenario B (Table S5 in Multimedia Appendix 1), Sonnet 4.5 already achieved lower JSD than GPT-5.2 in the zero-shot condition (0.1383 vs 0.1887), and CoT produced no additional benefit for Sonnet 4.5 (#JSD=0); its responses were identical under the 2 conditions, with a 23/77 split between options A and B. GPT-5.2 improved moderately under CoT (18% JSD reduction, from 0.1887 to 0.1539), converging toward a 50/50 A-B split, but Sonnet 4.5’s zero-shot distribution nonetheless remained closer to the human preference for option B (A=23.1%, B=76.9%; human A=23.1%, B=49.7%).

Crucially, neither model recovered the tail of the human response distribution on perception items: humans selected options C or D on 23% of DS3 perception-based responses, whereas both models concentrated exclusively on A and B. CoT could also induce high-level recategorization rather than simple refinement; for example, Sonnet 4.5’s majority selection for Q6 reversed from option B (77%) under zero-shot to option A (77%) under CoT, indicating that explicit reasoning can fundamentally alter the model’s interpretive direction rather than incrementally adjust its confidence.

### Effect of Discharge Summary Type on the Education Gap

Given Sonnet 4.5’s overall education gap, we next examined whether discharge summary characteristics modulated the size and direction of that gap. Per-discharge summary alignment results are reported in Table S6 in Multimedia Appendix 1. Figures 4-6 provide complementary views of how education-stratified alignment varied across summaries and question types. In Figure 4, the perception-item panel shows the largest education gap on DS1, the short-and-difficult summary, where the gap reaches +1.50, the largest cell-level disparity in the analysis. By contrast, the information-item panel shows the DS1 gap as only a mild reversal (Δ=−0.13), indicating roughly comparable alignment for the low- and high-education subgroups on factual-recall items.

**Figure 4.**
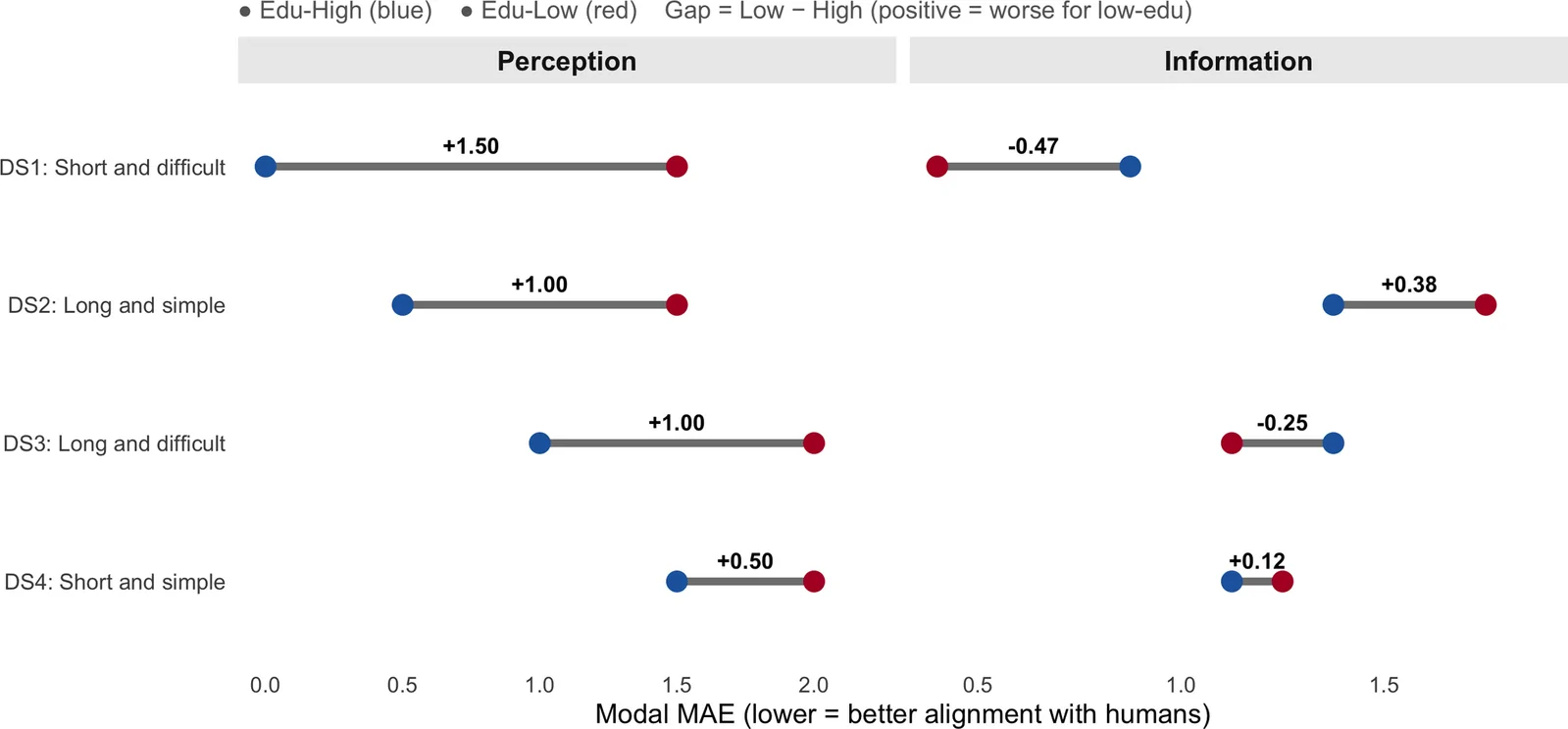
Education gap by discharge summary type for Sonnet 4.5. Each row represents one discharge summary type. Blue dots indicate the modal MAE for the high-education subgroup and red dots for the low-education subgroup; the length of the connecting line corresponds to the education gap (∆=Low − high). DS1 (short and difficult) exhibited the largest perception gap (+1.50). DS: discharge summary; MAE: mean absolute error.

**Figure 5.**
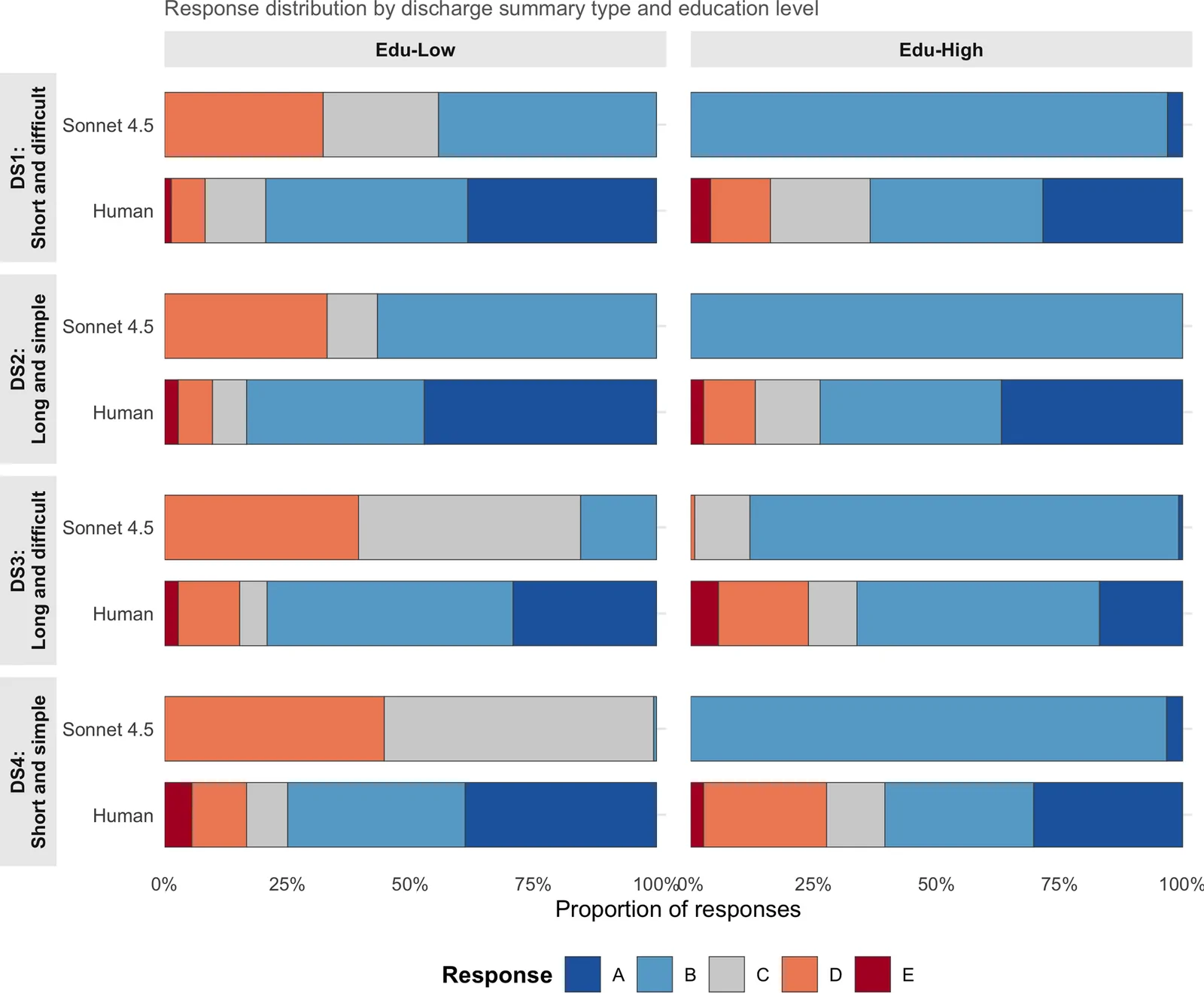
Response distributions for perception-based questions, comparing Sonnet 4.5 against human selections. Rows represent the 4 discharge summary types and columns the low- and high-education subgroups. DS: discharge summary.

**Figure 6.**
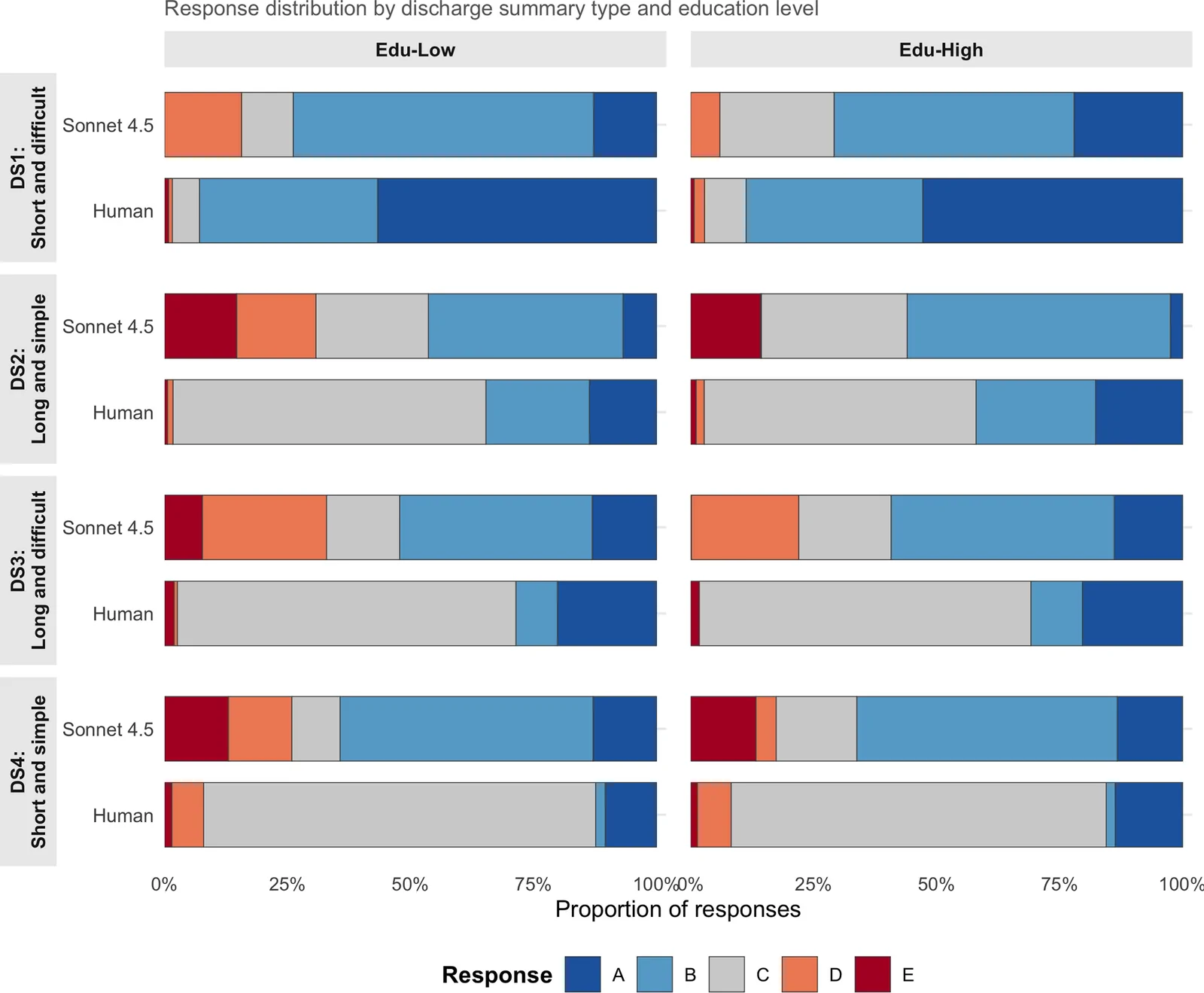
Response distributions for information-based questions, comparing Sonnet 4.5 against human selection. The layout matches Figure 5. DS: discharge summary.

On perception-based questions, the education gap varied substantially by discharge summary type, as shown in Figure 5. Each horizontal stacked bar shows the proportion of responses from A (dark blue, most positive) to E (dark red, most negative). For DS1 in the low-education condition, the human distribution concentrates on options A and B, whereas LLM shifts mass toward options C and D, illustrating the model’s tendency to assume lower clarity and confidence for low-education personas. The largest gap occurred on DS1: Sonnet 4.5 achieved a modal MAE of 0.00 for the high-education subgroup but 1.50 for the low-education subgroup, yielding a gap of +1.50. DS2, the long-and-simple summary, showed the second-largest perception-item gap (+1.00). DS3 and DS4 showed more moderate gaps (+0.50 each), although the absolute low-education modal MAE remained high, ranging from 1.50 to 2.00.

Per-question inspection suggested a consistent mechanism. When conditioned on low-education personas, Sonnet 4.5 repeatedly selected option B or C for Q1, which asked about overall clarity, and option D for Q10, which asked about confidence and comfort, regardless of discharge summary type. Low-education human respondents, however, selected A (“very clear” or “very confident”) for Q1 on 3 of the 4 summaries and B for Q10 on all 4 summaries. Thus, the model’s low-education persona responses were systematically shifted toward lower perceived clarity and confidence than the corresponding human responses. This pattern suggests that Sonnet 4.5 encoded a stronger association between lower education and difficulty understanding or feeling confident about the discharge summary than was observed in the human data. In contrast, when conditioned on high-education personas, the model’s predominantly option B selections more closely matched the high-education human modes, producing low high-education modal MAE and thereby widening the education gap on perception items.

On information-based questions, the DS1 education gap is small, as shown in Figure 6 (Δ=−0.13 modal MAE; −0.07 EAD): the low-education persona matched its human counterpart only slightly better than the high-education persona matched its own. DS2, the long-and-simple summary, showed a small positive information-item education gap (+0.25 modal MAE), and DS3 and DS4 showed corresponding gaps of +0.25 and −0.25; no discharge summary stands out as a strong driver of education-related information-gap behavior.

Two broader document-level patterns emerged. First, medical complexity appeared to widen the perception-item education gap most strongly when the discharge summary was short. DS1, the short-and-difficult summary, produced the largest perception-item gap (+1.50), whereas DS4, the short-and-simple summary, produced a smaller gap (+0.50). This contrast suggests that, when a summary was both brief and medically complex, Sonnet 4.5’s low-education persona responses were shifted toward lower perceived clarity and confidence, even though low-education human respondents did not show a correspondingly large decrease in reported clarity.

Second, document length does not appear to widen the information-item education gap. The 4 per-DS information-item education gaps lie within |·|≤0.25 and do not order by length or by complexity.

Finding 3 (Discharge summary effect [low-education personas]): Alignment for low-education personas is primarily driven by short discharge summaries (DS1 and DS4), in which the reduced textual load elicits more consistent and predictable human responses that the persona-conditioned LLM can reproduce.

Finding 4 (Discharge summary effect [high-education personas]): Alignment for high-education personas is primarily driven by easy-to-understand discharge summaries (DS2 and DS4) and appears largely insensitive to document length. Simpler language, rather than shorter, is the discharge summary characteristic most important for faithful simulation of the high-education subgroup.

### Misalignment Analysis

Although aggregate LLM response patterns were often close to human response patterns, specific items showed substantial misalignment. We analyzed these discrepancies for Sonnet 4.5, which showed the largest education-related equity gap and the poorest aggregate alignment on information-based questions among the evaluated models (modal MAE=0.90; EAD=0.92). We examined all 32 information-based questions (4 discharge summaries ×8 questions), comparing the pooled modal responses of Sonnet 4.5 and human participants. The overall distribution of per-question modal distances is summarized in Table S8 in Multimedia Appendix 1, and the 7 worst-aligned questions are presented in Figure 7 and detailed in Table S9 in Multimedia Appendix 1. The left panel in Figure 7 shows human distributions, and the right panel shows LLM distributions. Colors encode ordinal responses from A (dark blue, most positive) to E (dark red, most negative). The ∆ annotation indicates the ordinal gap between modal responses. DS2-Q6 (“when to call the doctor’') exhibits the single largest gap (∆=4): the model places all its mass on E (“all of the above”), whereas humans select specific symptoms (A: 34%, B: 33%, D: 32%). Discharge plan coverage (Q9 across DS1, DS2, and DS3) and other-prescription identification (Q5 on DS3 and DS4) account for the remaining 6 ∆≥2 gaps.

**Figure 7.**
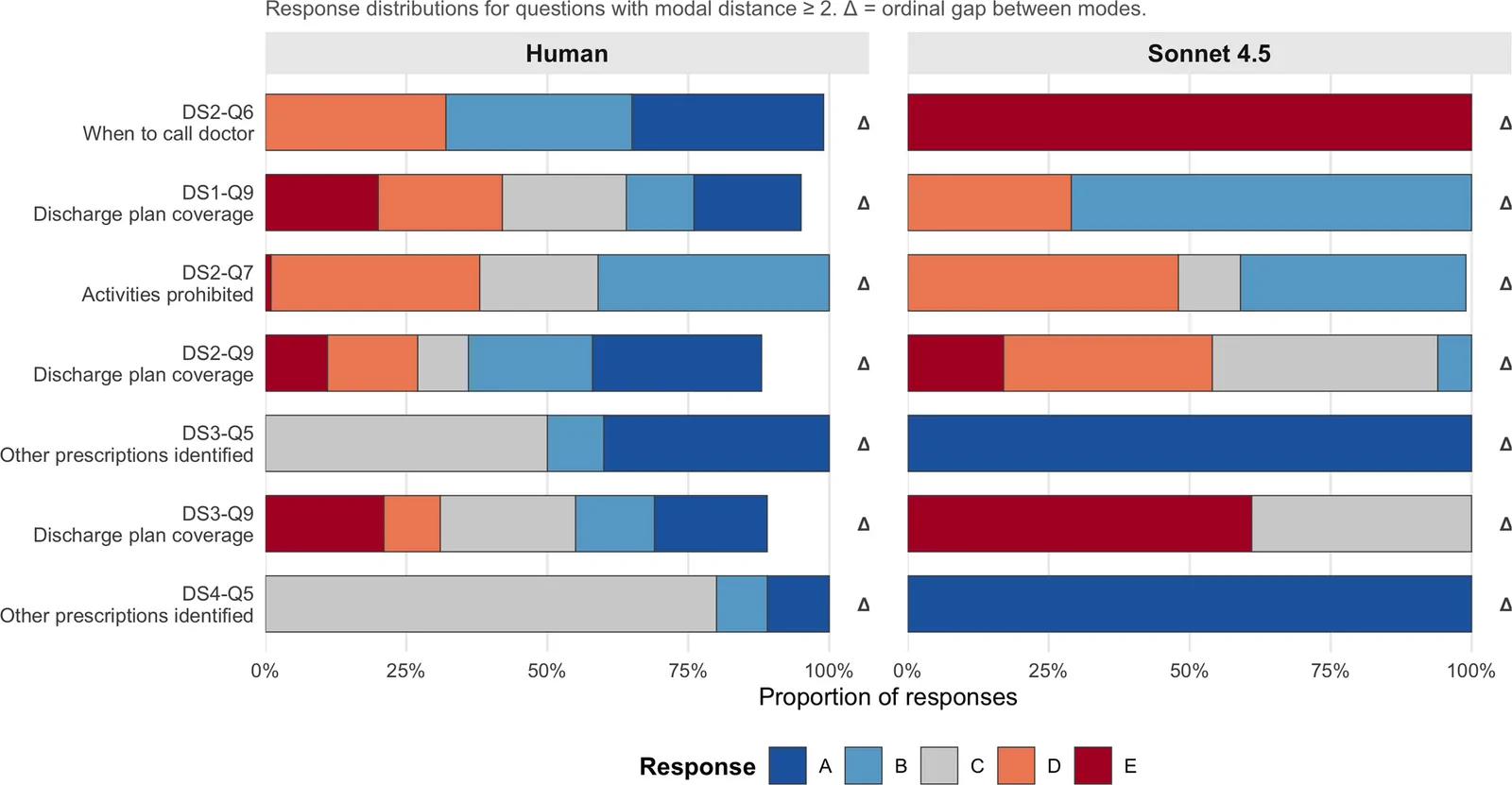
Response distributions for the 7 worst-aligned information questions (modal distance ≥2), comparing Sonnet 4.5 against human selection. DS: discharge summary.

Four recurring misalignment patterns emerged. First, factual-extraction questions (Q2-Q4) were the most pervasive source of error. On DS2, DS3, and DS4, Sonnet 4.5 selected option B (“Yes, identified”) with near-100% probability, whereas human respondents predominantly selected option C (“Not provided”) on the same questions, with human selection rates of 54%‐87%. We refer to this pattern as identification optimism: the model treated information as identifiable when human respondents generally did not. This pattern is consistent with the model using textual or medical cues that were either ambiguous or not salient to many lay readers.

Second, the single largest gap (modal distance 4) occurred on DS2-Q6, which asks which symptoms should prompt a call to the doctor. The model placed 100% of its mass on option E (“All of the above”), whereas humans almost never picked the catch-all and instead distributed their selections across the specific symptoms emphasized in the discharge summary (A: fever >101=34%; B: chest pain=33%; D: shortness of breath=32%). The model treats this check-all-that-apply item as if the safest answer is to flag every option, whereas human respondents identify the particular symptoms the document warns about. This is the only item in the information block with *Δ*≥3.

Third, the other-prescription identification item (Q5) showed a directionally reversed pattern across discharge summaries. On DS3 and DS4, 50%‐80% of humans selected option C (“not provided”), whereas the model placed 100% of its mass on option A (“identified”); on DS1, the human and model modes coincide on option C. The model thus overclaims that additional prescriptions are documented in the long, dense discharge summaries while patients on the same documents report the opposite.

Fourth, the discharge-plan-coverage item (Q9) is a check-all-that-apply question listing 7 components of the discharge plan (medication schedule, follow-up, symptoms, diet, activity, other, and none). Three of the 4 discharge summaries (DS1, DS2, and DS3) yielded a distance-2 gap because the model concentrated on 1 or 2 components (for example, DS1: B+D=100%; DS3: C+E=100%), whereas humans reported broader coverage spanning 4 or 5 components. The model appears to treat the item as single-best-answer rather than recognizing its check-all-that-apply nature, producing systematically narrower coverage profiles than the human-reported ones.

Overall, 11 of the 32 information-based questions (34%) achieved perfect modal alignment. The dominant failure mode was the identification-optimism pattern described above, especially on DS2-DS4, where key information was absent, indirect, or ambiguously presented. These results suggest that Sonnet 4.5 may rely on textual or clinical cues that allow it to infer an answer even when human respondents more often report that the relevant information is not provided or that they do not know.

The underselection of uncertainty options (“Not provided” and “I don’t know”) should be interpreted considering the prompting design, not attributed exclusively to model-internal bias. Our prompt instructed the model to output only a final answer letter; this forced-choice format provides no mechanism to hedge, defer, or mark low confidence. The CoT sensitivity analysis partially supports this interpretation: allowing a brief reasoning trace modestly redistributed probability mass across options on information-based items (Table S4 in Multimedia Appendix 1) but did not recover the full human uncertainty distribution. We therefore interpret the forced-choice format as a structural confound that contributes to, but does not fully explain, the identification-optimism pattern, and return to this limitation in the “Discussion” section.

Finding 5 (Response homogeneity): LLMs more closely approximate subgroups whose human responses are relatively homogeneous. Subgroups and item types with high within-group response entropy, especially perception-based items on longer or more ambiguous discharge summaries, were consistently harder to model. In these settings, persona-conditioned LLM responses tended to concentrate probability mass on 1 or 2 adjacent options rather than reproducing the broader dispersion observed in human responses.

Finding 6 (Comprehension overestimation): All 4 models tended to overestimate how easily patients could extract information from discharge summaries, particularly longer ones. On factual-extraction items (Q2-Q4), Sonnet 4.5 selected the “identified” option with near-100% probability on DS2, DS3, and DS4, whereas human respondents predominantly reported that the corresponding information was not clearly provided or that they did not know. The model assigned little probability mass to difficulty-indicating or uncertainty response options that general-population participants selected frequently.

## Discussion

### Principal Findings

This study provides a comprehensive evaluation of whether contemporary LLMs can faithfully simulate patient perspectives on hospital discharge summaries, and of whether that simulation is equitable across demographic subgroups. Using a single standardized decoding protocol across 4 mainstream models, we benchmarked persona-conditioned response distributions against those of 99 general-population participants recruited through Prolific. The resulting pattern of alignment and misalignment directly addresses the 3 hypotheses prespecified in the “Introduction” section.

### H1 (Task-Type): Supported

Across all 4 models, persona-conditioned alignment was consistently closer for information-based items than for perception-based items, both in the modal-response metric (Table 2) and in the full-distribution metric (Table 3). This gap between question types was larger than any cross-model difference, indicating that the question type is the dominant determinant of simulation fidelity. Factual-recall items appear to be amenable to LLM simulation because the underlying “correct” answer is constrained by the text of the summary, whereas perception items elicit subjective comprehension judgments that vary between real humans and are not recoverable from the text alone.

### H2 (Demographic-Equity): Partially Supported

Persona-based alignment was most reliable when stratified by education and substantially less reliable when stratified by gender, doctor visits, or ED visits, consistent with the first half of H2. The second half: at least one model would show an equity gap exceeding the human split-half noise floor, was also supported: Sonnet 4.5 exhibited a low-vs-high education gap on perception modal MAE (low 1.62 vs high 0.75), more than twice the corresponding split-half baseline (0.39 low, 0.64 high) and therefore not attributable to within-group noise. GPT-4.1 showed the largest gender gap on perception modal MAE, and Opus 4.6 was the most equitable model on both axes. EAD gender gaps were uniformly small across models, indicating that gender-related differences arise from mode-switching sensitivity at the item level rather than systematic distributional bias.

### H3 (Document-Dependence): Supported (Perception) and Not Supported (Information)

The magnitude and direction of the Sonnet 4.5 education gap on perception items were strongly modulated by discharge summary characteristics. On the short-and-difficult summary (DS1), the perception gap reached +1.50, the single largest cell-level disparity in the analysis. The information-item education gap on DS1 is only −0.13, and per-DS information-item gaps are uniformly within |·|≤0.25 with no clean ordering by length or by complexity. Medical complexity therefore amplifies the perception gap when documents are short; document length does not analogously amplify the information gap when documents are simple. Persona effects on perception items therefore appear to interact with document structure, while persona effects on information items appear largely independent of it.

Beyond the 3 hypotheses, a sensitivity analysis showed that CoT prompting produced a 66% reduction in JSD for Sonnet 4.5 on information-based items on DS1 (from 0.0847 to 0.0289) but essentially no change for GPT-5.2 on the same items. On perception questions, CoT did not recover the tail of the human distribution for either model: humans selected options C or D on 23% of DS3 perception-based responses, whereas both models concentrated entirely on options A and B regardless of prompting condition. The prompt-level intervention that matters most is therefore model- and task-specific rather than universally beneficial.

### Why Education Is the Tractable Axis

Our results suggest that persona-based simulation is reliable where real human responses are themselves homogeneous, and brittle where they are diverse (Finding 5). Within the educational attainment stratification, low-education and high-education subgroups produced near-identical aggregate response distributions (Table S1 in Multimedia Appendix 1), with pairwise agreement of 54.8% and 52.3%, respectively and agreement on the plurality response for 33 of 40 (82.5%). The persona-conditioned LLM can therefore hit the human mode by defaulting to a plausible general-population answer, which explains why education yields the smallest overall alignment gaps. By contrast, gender subgroups show larger within-group disagreement on perception items, which no model reproduces: the 4 evaluated LLMs concentrated probability mass on 1 or 2 adjacent response options, failing to cover the tail of the human distribution. The implication is that current LLMs are adept at simulating the modal member of a subgroup but not its heterogeneity, which matters most for perception items and for subgroups defined by behavioral rather than educational attributes.

### Where Simulation Fails: Identification Optimism and Forced-Choice Uncertainty

Beyond the equity patterns, a recurring misalignment mode was the tendency of Sonnet 4.5 to overselect the “identified” option on factual-extraction questions (Q2-Q4) on DS2, DS3, and DS4, while human respondents selected “Not provided” or “I don’t know” at frequencies of 54% to 87% (Finding 6). We interpret this identification optimism as a product of 2 interacting mechanisms. First, the model’s superior text-comprehension capability allows it to extract information that a general reader may not locate, so the same document reads as more informative to the model than to the patient it is impersonating. Second, the forced-choice prompt (output only a letter, with no explanation) prohibits the model from hedging or marking low confidence, structurally suppressing uncertainty expression. The CoT sensitivity analysis partially supports this interpretation: a brief reasoning trace modestly redistributed mass but did not recover the human uncertainty distribution. We therefore regard the forced-choice prompt as a structural confound that contributes to the underselection of uncertainty options; a parallel observation is that training and evaluation pipelines implicitly reward confident guesses over admissions of uncertainty [41]. A complementary exact-match analysis sharpens this picture: on the confidence-axis items Q2-Q5, Sonnet 4.5’s exact-match alignment with the human mode is 24% versus 83% for Opus 4.6. The lowest of the 4 evaluated models on precisely the items where the ordinal metric counts the bias as a 1-step “near miss.” On information items, Opus 4.6 achieves the highest exact-match alignment with the human mode and Sonnet 4.5 the lowest.

### Implications for Patient-Centered Communication and for LLM Evaluation

A well-calibrated LLM simulator could support scalable pretesting of patient-facing communications, reducing the need for large-scale human panels at every revision cycle [11,12]. Our results highlight 3 considerations for implementing this vision in practice. First, persona simulation is most reliable where it is least necessary, on subgroups whose responses are already homogeneous, and is least reliable on subgroups whose heterogeneous responses are precisely what readability testing aims to capture. Second, model choice is a crucial design decision: among the 4 evaluated models, there are significant differences in fairness characteristics. Therefore, the statement “LLMs simulate patients” cannot be regarded as independent of the model. Third, LLM character simulation should serve as a supplement to human assessment rather than a replacement; it may be applicable for initial screening or cost-effective initial iterations, but production-level decisions for patient communication should still continue to involve real participants from the target population.

The equity pattern we observe also has a direct responsible-AI interpretation. If LLMs systematically reproduce the comprehension patterns of privileged subgroups (high education, male, and low health care use) while underrepresenting marginalized ones, their adoption without subgroup-stratified evaluation would silently encode existing health-literacy gaps into the next generation of patient-facing documents, a concern aligned with prior evidence of demographic bias in LLM medical content [15,42,43] and with the long-standing underrepresentation of nonmale, non–highly-educated participants in medical research [44].

### Limitations

Several limitations temper the interpretation of these findings. First, although we enforced a single standardized decoding protocol across the 4 tested models (temperature 1.0; top p 1.0; frequency penalty and presence penalty 0.0; 10 independent iterations per persona-question cell), the 4 models themselves differ in training data composition and Reinforcement Learning from Human Feedback (RLHF) recipes, so the equity characteristics we reported for the models should not be extrapolated uncritically to future model releases or to open-source models not tested here.

Second, the LLM persona prompt varied age (25, 45, and 65 years) and race and ethnicity (White, Black, Hispanic, and Asian), but these axes were not stratified in the alignment analysis because the human cohort was unbalanced along them: only 6 respondents fell in the 55-and-older bracket (vs 21 in 18‐24, 38 in 25‐34, and 23 each in 35‐44 and 45‐54), and the smaller race and ethnicity subgroups had similarly limited cell sizes. The equity-gap analyses therefore focus on education and gender (primary) and visit frequencies (secondary). Pooling across age and race and ethnicity may mask demographic-specific patterns and represents a confound to be disentangled in future work. Also, the persona prompt uses nonstandard grammatical formulations (eg, “you from {ethnicity} race”) that were held constant across all personas; this phrasing cannot account for between-subgroup differences but may affect the absolute level of human-LLM alignment by associating the prompt with a reduced linguistic register.

Third, our instrument is a single-turn, forced-choice, 10-item questionnaire rather than a free-text comprehension task. This design supports systematic, reproducible benchmarking across demographic subgroups, but it also operationalizes “patient perspective” as a behavioral response tendency rather than as deeper semantic comprehension. As discussed above, the forced-choice format additionally provides no mechanism for the model to hedge, defer, or explicitly signal low confidence, which likely contributes to the observed underselection of “Not provided” and “I don’t know” options alongside any model-internal training bias; we have not fully disentangled the two.

Fourth, the 4 discharge summaries were drawn from MIMIC-IV and were selected to instantiate a 2×2 design that crosses length (short vs long) with medical complexity (simple vs difficult). With n=1 document per cell, document-specific content effects cannot be fully disentangled from category-level effects, and complexity was operationally defined using qualitative criteria (density of medical abbreviations, jargon, and sentence length) rather than validated readability instruments such as Flesch-Kincaid [45] or the Simplified Measure of Gobbledygook (SMOG) [46]. Results may also differ for outpatient documents, non-English documents, or clinical contexts outside intensive care unit (ICU) discharge.

Fifth, the human reference cohort consists of online crowd-workers recruited through Prolific, not patients actively undergoing transitions in care. Healthy participants in a controlled online environment do not fully reproduce the cognitive load, psychological stress, or physical symptoms of patients during or immediately after an ICU stay. This study therefore establishes a general-population comprehension benchmark against which model-human alignment can be evaluated, and should be read as a prerequisite to, not a substitute for, clinical validation with the patient populations that the technology is ultimately intended to serve.

In addition, the synthetic cohort was generated using a full-factorial persona grid, whereas the human cohort reflects a naturally occurring and uneven joint demographic distribution. Consequently, aggregate synthetic-human comparisons should be interpreted cautiously; subgroup-stratified comparisons within matched demographic groups provide the most appropriate assessment of alignment. Additionally, the high-frequency ER-visit stratum contained only 3 participants, which precluded a robust statistical comparison between this subpopulation and LLM outputs. Our subgroup findings for health care use personas may therefore underrepresent the most frequent health care users; likewise, the nonbinary gender stratum (n=6) is too small for stable entropy or alignment estimates, and we have reported values for this group only as supplementary materials reference points rather than as a basis for substantive conclusions.

Finally, statistical inference in this paper is based on a nonparametric human split-half baseline; per-cell bootstrap CIs and permutation-test *P* values on every paired equity gap remain a priority for follow-up work.

### Comparison to Prior Work

ICL enables LLMs to adapt to tasks via prompts alone, without modifying their underlying parameters [4,22-24]. Prompts define scenarios or pose questions to steer the model toward contextually appropriate responses [25].

LLMs can perform effectively even in zero-shot settings, demonstrating strong capabilities without example-based prompts [47], and this adaptability extends to reasoning and problem-solving without explicit training examples [26,27]. Prompt engineering is therefore central to maximizing the effectiveness of ICL [30,31]: prompt structure and content can substantially influence model outputs [48]. Persona-based prompts in particular guide LLMs to assume specific roles or perspectives [28,29] and can affect tone, style, and factuality [32], including eliciting more truthful outputs by framing queries through personas [33]; adversarial personas can conversely steer models toward harmful behaviors [49]. More broadly, prompt framing can induce shifts in personality, emotional tone, and other human-like traits [17,50], supporting the simulation of complex human behaviors and demographically conditioned responses.

LLMs have been widely applied in the medical domain, including research, medical education, and clinical tasks. They have shown promise in generating and summarizing patient discharge instructions [51,52]. For example, Ng et al [53] demonstrated more efficient workflows when clinicians generated discharge summaries using a retrieval-augmented generation (RAG) AI-assisted tool. AI tools are increasingly integrated into health care professionals’ daily workflows, with studies such as Garcia et al [12] reporting that ChatGPT (OpenAI) use may reduce burnout by as much as 20%.

Despite these strengths, LLMs are susceptible to biases inherited from their training data and reinforced by human feedback [13,54]. Such biases can surface in outputs and may be amplified under certain prompting strategies, including persona-based approaches [14-16]. This raises critical challenges for ensuring fairness and neutrality in model responses [17,18]. Moreover, the adoption of LLM-based systems may exacerbate automation bias, as their outputs, delivered in fluent, human-like language, are often perceived as more relatable, credible, and trustworthy than warranted [21]. A call has also been announced regarding the need for AI disclosure guidelines in patient communication [55].

### Future Directions

Beyond the specific research extensions enumerated in the “Discussion” section, 3 broader directions are needed before LLM-based persona simulation can be responsibly embedded in patient-centered health care communication. The first is the development of patient population–aware medical LLMs, trained or fine-tuned on data contributed by minority and marginalized communities with the explicit objective of reproducing in-group self-perceptions rather than out-group stereotypes [15,43]. The equity patterns observed in our study, in which simulation fidelity is highest for already advantaged subgroups and lowest for those most in need of readability interventions, underscore that off-the-shelf models are unlikely to close this gap without targeted, community-grounded data collection.

The second is integration with ongoing patient feedback in deployed clinical workflows rather than as one-time predeployment screening. An LLM clarity assessor that is calibrated once and then frozen will eventually drift from the populations it serves; pipelines that incorporate longitudinal patient feedback, for example, from postdischarge follow-up calls or patient portals, could continuously recalibrate automated clarity scores against the comprehension patterns of the specific populations each institution serves, while also surfacing demographic subgroups for whom the tool should not be used without human oversight.

The third is a governance norm that treats subgroup-stratified evaluation and model-choice disclosure as prerequisites for deployment. Our results show that equity characteristics differ substantially across 4 contemporary models and vary with document structure; a clinical tool that reports only an aggregate readability score obscures exactly the subgroup-level disparities that make this technology consequential for health equity. Institutional review, professional-society guidelines, and emerging AI-in-health-care regulation should, in our view, require that patient-facing LLM tools report per-subgroup performance and that deployment decisions document which demographic groups the selected model has been shown to serve equitably.

By addressing these broader directions alongside the immediate research extensions identified above, we hope to move toward automated patient-centered communication that is not only scalable and effective but also equitable, broadening accessibility for patient populations that have historically been underrepresented in both medical research and in the training data of the models that now shape their care.

### Conclusions

This study evaluated whether contemporary LLMs can simulate patient perspectives on hospital discharge summaries when conditioned on personas defined by education, gender, and health care use. Compared with responses from a 99-participant general-population cohort, persona-conditioned LLM responses aligned more closely with humans on information-based items than on perception-based items, and alignment was most consistent for education-stratified personas. However, simulation fidelity varied across models, demographic subgroups, and discharge summary characteristics, indicating that persona-based alignment is neither uniform nor model-agnostic.

Overall, contemporary LLMs can approximate the modal response of a subgroup but remain limited in capturing the heterogeneity of patient understanding, particularly for subjective perceptions and uncertainty. Persona-based LLM simulation should therefore be used as a scalable assistive tool for identifying potential clarity risks in draft discharge summaries, rather than as a substitute for human patient feedback or clinical validation. Before patient-facing deployment, such systems require institutional validation, human oversight, subgroup-stratified evaluation, and careful model selection.

## Supplementary material

10.2196/89003 Multimedia Appendix 1 Prompt template and examples, discharge summary samples, survey questions, and supplementary tables.

## Abbreviations

CoT: chain-of-thought
EAD: expected absolute distance
ER: emergency room
ICL: in-context learning
ICU: intensive care unit
IRB: Institutional Review Board
JSD: Jensen-Shannon divergence
LLM: large language model
MAE: mean absolute error
MIMIC-IV: Medical Information Mart for Intensive Care
RAG: retrieval-augmented generation
RLHF: Reinforcement Learning from Human Feedback
SMOG: Simplified Measure of Gobbledygook
