# Supplementary material for: Large Language Models’ Simulation of Patient Comprehension of Discharge Summaries: Multimodel Evaluation Study
Source: J Med Internet Res. 2026 Oct 22;28:e89003. doi: 10.2196/89003 (PMC13690339; doi:10.2196/89003)
Supplement: Supplementary file 1 [file jmir-v28-e89003-s001.docx]

**Multimedia Appendix**

# 1.0 Prompt Template and Example

You are a {age} {gender} with {education} education level, you from {ethnicity} race^[1]^, you visit doctor {doctor_visit}, and visit emergency room {er_visit_frequency}.

You will be reading a Discharge Summary written by a clinician for a patient. After reading the Discharge Summary, I will ask you a question. Your job is to think of someone with your background and choose the correct answer by selecting the letter of the answer (e.g., A, B, C, etc.). You should only output the letter with your final answer without any explanation.

Here is the Discharge Summary: “You were admitted and found to have an ulcer in the duodenum. To help this heal, we are prescribing new medications (pantoprazole). Please be sure to take this until you are seen in follow-up.”

Now, answer the following question: Question: Please rate your understanding level of this discharge instruction. A. Very clear B. Somewhat clear C. Not clear at all Your task: Only respond with the letter of the answer that best reflects your understanding.

Persona Example 1. You are a 25, male with low education level, you from Black race, you visit doctor low and visit emergency room low.

Persona Example 2. You are a 65, female with high education level, you from Hispanic race, you visit doctor high and visit emergency room low.

# 2.0  Discharge Summary Samples

DS1 You were admitted and found to have an ulcer in the duodenum. To help this heal, we are prescribing new medications (pantoprazole). Please be sure to take this until you are seen in follow-up.

DS2 Call Dr. xxx if experience:

-Take stool softeners with narcotics

-Fever *>* 101 or chills

-Increased shortness of breath or cough

-Chest pain

-You may shower. No swimming for 4 weeks -No driving while taking narcotics.

DS3 Keep splint/dressing on until follow-up

Keep splint clean & dry at all times

Follow up with Dr. xxx in xxx days

Wean off of narcotics Take aspirin for 2 weeks Physical Therapy:

NWB left lower extremity

Leave splint on until follow-up

DS4 Ok to shower today but wear tegaderm dressing over the drain site. No heavy lifting. Return to ED for anything that concerns you.

# 3.0 Survey Questions^[2]^*

## 3.1 Information-based Questions

Q2 Do you know the name of all your medications? (If yes, please type.) (A) Yes (B) I don’t know (C) Not provided

Q3 Do you know your diagnosis? (If yes, please type.) (A) Yes (B) I don’t know (C) Not provided

Q4 Do you know the common side effects of all your medications? (If yes, please type.) (A) Yes (B) I don’t know (C) Not provided

Q5 Are there other prescriptions given besides the medication? (If yes, please type.) (A) Yes (B) I don’t know (C) Not provided

Q6 [DS1/DS3/DS4] Do you know what kind of condition you have mentioned in the discharge instructions? [A] Stomach disease [B] Ulcer in the duodenum [C] Wearing Tegaderm [D] Keep splint [E] I don’t know

[DS2] What are some situations that you have to call Dr.  ___? [A] Fever > 101 [B] Chest Pain [C] Stomach Pain/diarrhea [D] Shorten of breath [E] All of the above [F] I don't know

Q7 [DS1/DS4] Do you know what kind of treatment you need to follow based on the discharge instructions? [A] Nothing [B] Take a new medication [C] See a doctor again [D] I don’t know

[DS2] Do you know what kind of treatment you need to follow based on the discharge instructions? [A] Nothing [B] Swimming in 4 weeks [C] Consuming narcotics without stool softeners [D] Driving while under influence of narcotics [E] Consuming Alcohol [F] I don't know

[DS3] Do you know what kind of treatment you need to follow based on the discharge instructions? [A] Take a new medication [B] See a doctor again [C] Non-weight-bearing left lower extremity [D] Nothing [E] I don't know

Q8 Are there any activities or foods you need to avoid? [A] Avoid fruit [B] Avoid strenuous exercise [C] Others [D] I don’t know

Q9 Is there anything about your discharge instructions that is unclear or worrying you? [A] Medication schedule [B] Follow-up appointments [C] Symptoms to watch for [D] Dietary restrictions [E] Activity limitations [F] Other, please specify. [G] No, it’s very clear

## 3.2 Perception-based Questions

Q1 Please rate your understanding level of this discharge instruction. (A) Very clear. (B) Somewhat clear. (C) Not clear at all.

Q10 Please rate the difficulty in understanding this discharge instruction. (A) Extremely easy (B) Somewhat easy (C) Neither easy nor difficult (D) Somewhat difficult (E) Extremely difficult

# 4.0 Supplementary Tables

This section contains supporting tables referenced from the Results sections. All tables use the “Supplementary Table” prefix in the main text and carry an appendix-style table number (e.g., Table S1, S2, ...).

## 4.1 Human Response Distributions and Pairwise Agreement

Table S1: Human overall response distribution by gender. Columns A–D are percentages of mapped responses pooled across all questions; $H^{\left( A\text{-}D \right)}$and $H_{n}^{\left( A\text{-}D \right)}$ are Shannon entropy on the pooled A–D distribution, $H_{n}^{\left( A\text{-}D \right)}$ denotes normalized entropy (maximum 2.0 for four response categories). $\bar{H}_{n}$ (per-Q) is the per-question normalized entropy, defined as $\bar{H}_{n}=\left( 1/Q \right)\sum_{q} H_{q}/{\log_{2} k}_{q}$, with $k_{q}$ for each question $q$ with distribution $p(q)$ over its actual option set of size $k_{q}$, compute $H_{q}$, then average $H_{q}/{\log_{2} k}_{q}$ across questions.

| **Gender** | **N** | **A** | **B** | **C** | **D** | $H^{\left( A\text{-}D \right)}$ | $H_{n}^{\left( A\text{-}D \right)}$ | $\bar{H}_{n}$(per-Q) |
| --- | --- | --- | --- | --- | --- | --- | --- | --- |
| Male | 35 | 26.9% | 28.1% | 33.1% | 11.9% | 1.918 | 0.959 | 0.485 |
| Female | 58 | 24.9% | 25.6% | 37.4% | 12.1% | 1.902 | 0.951 | 0.473 |
| Non-binary* | 6 | 26.9% | 25.1% | 38.6% | 9.4% | 1.860 | 0.930 | 0.353 |

$H_{n}^{\left( A\text{-}D \right)}=H/\log_{2} 4$. Responses mapped to E, F, or G are excluded from the pooled statistic (1.5–2.6% of responses per subgroup). $\bar{H}_{n}\in\left[ 0,1 \right]$ regardless of $k_{q}$, with $k_{q}\in\left\{ 2,3,4,5,7 \right\}$ across the 40 questions (e.g., 3 for Q1, 5 for Q10, 7 for Q9). The substantial gap between $H_{n}^{\left( A\text{-}D \right)}\approx0.95$ and $\bar{H}_{n}(per-Q)\approx0.48$ for Male and Female indicates high within-question consensus; the pooled marginal diversity is driven by between-question variation in modal answer, not within-question disagreement. Multi-select answers (DS2 Q6/Q7 and Q9 across all DS) are split so that each chosen option contributes one vote to the per-question distribution; the per-question option count k_q used for normalization is the actual number of options presented for that item.

* For the non-binary group, entropy estimates are unreliable due to the small sample size (N=6).

| **Education** | **N** | **A** | **B** | **C** | **D** | $H^{\left( A\text{-}D \right)}$ | $H_{n}^{\left( A\text{-}D \right)}$ | $\bar{H}_{n}$(per-Q) |
| --- | --- | --- | --- | --- | --- | --- | --- | --- |
| Low | 48 | 26.0% | 26.6% | 36.4% | 11.0% | 1.895 | 0.947 | 0.489 |
| High | 50 | 25.4% | 26.2% | 35.6% | 12.7% | 1.918 | 0.959 | 0.499 |

Low and High differ by $\Delta H_{n}^{\left( A\text{-}D \right)}=0.012$ and $\Delta\bar{H}_{n}=0.010$, essentially indistinguishable under either statistic, and the absolute level for $\bar{H}_{n}$(per-Q) near 0.5 reflects human education level has negligible influence on within-group response diversity.

Table S2: Within-group pairwise agreement by gender and education level, including all response options A-G.

| **Gender** | | | **N** | | **Overall** | | **Information** | | **Perception** | |
| --- | --- | --- | --- | --- | --- | --- | --- | --- | --- | --- |
| Male | | | 35 | | 50.8% | | 53.7% | | 39.3% | |
| Female | | | 58 | | 55.0% | | 59.5% | | 36.9% | |
| Non-binary^∗^ | | | 6 | | 60.9% | | 65.0% | | 44.6% | |
|  | **Education** | **N** | | **Overall** | | **Information** | | **Perception** | |  |
|  | Low | 48 | | 54.8% | | 58.9% | | 38.2% | |  |
|  | High | 50 | | 52.3% | | 56.0% | | 37.2% | |  |

Pairwise agreement is the probability that two randomly chosen respondents in the subgroup selected the same response, averaged across questions. ^∗^ Estimates for the non-binary group are unreliable due to small sample size.

## 4.2 Education Robustness Check

Table S3: Alignment rate for the low-education LLM subgroup against two human low-education reference definitions.

**Model**

**Information**

**Perception**

|  | Low | HS only | ∆ | Low | HS only | ∆ |
| --- | --- | --- | --- | --- | --- | --- |
| Sonnet 4.5 | 0.094 | 0.125 | +0.031 | 0.000 | 0.000 | 0.000 |
| Opus 4.6 | 0.406 | 0.438 | +0.031 | 0.125 | 0.125 | 0.000 |
| GPT-5.2 | 0.281 | 0.312 | +0.031 | 0.375 | 0.375 | 0.000 |
| GPT-4.1 | 0.531 | 0.500 | −0.031 | 0.250 | 0.250 | 0.000 |

∆ denotes the difference between the high-school-or-less reference and the merged low-education reference. The information denominator was 32 questions, and the perception denominator was 8 questions.

## 4.3 Chain-of-Thought Sensitivity Analysis

Table S4: Scenario A (information-based questions on DS1): overall response distributions and Jensen–Shannon divergence versus humans (13 iterations per question; *n* = 104 per cell).

| Model | Condition | JSD↓ | A | B | C | D | G |
| --- | --- | --- | --- | --- | --- | --- | --- |
| *Human reference* |  | — | .529 | .349 | .068 | .014 | — |
| Claude 4.5 Sonnet | Zero-shot | 0.0847 | .462 | .250 | .202 | .000 | .087 |
| Claude 4.5 Sonnet | CoT | **0.0289** | .538 | .250 | .192 | .019 | .000 |
| GPT-5.2 | Zero-shot | 0.0603 | .471 | .260 | .269 | .000 | .000 |
| GPT-5.2 | CoT | 0.0597 | .423 | .279 | .269 | .019 | .010 |

Chain-of-thought prompting produced a large improvement for Claude 4.5 Sonnet (∆JSD = −0*.*0558, a 66% reduction) but essentially no change for GPT-5.2 (∆JSD = −0*.*0006). Claude’s CoT distribution (A = 53.8%, B = 25.0%) was the closest of any model-condition pair to the human reference.

Table S5: Scenario B (perception-based questions on DS3): overall response distributions and Jensen–Shannon divergence versus humans (13 iterations per question; *n* = 26 per cell).

| Model | Condition | JSD↓ | A | B | C | D |
| --- | --- | --- | --- | --- | --- | --- |
| *Human reference* |  | — | .231 | .497 | .077 | .154 |
| Claude 4.5 Sonnet | Zero-shot | **0.1383** | .231 | .769 | .000 | .000 |
| Claude 4.5 Sonnet | CoT | **0.1383** | .231 | .769 | .000 | .000 |
| GPT-5.2 | Zero-shot | 0.1887 | .615 | .385 | .000 | .000 |
| GPT-5.2 | CoT | 0.1539 | .500 | .500 | .000 | .000 |

Neither model captured the full range of human responses. Options C and D, which together accounted for 23% of human responses, were selected by neither model under either prompting condition.

## 4.4 Per-Discharge-Summary Education Alignment for Sonnet 4.5

Table S6: Per-discharge-summary education-level alignment for Sonnet 4.5. For each discharge summary type, modal MAE and expected absolute distance (EAD) are reported for the low- and high-education subgroups, along with the education gap ( Δ = Low - High). Bold values indicate the largest gap within each metric block. Information items use a per-item ordinal scale whose maximum attainable distance varies from 2 (three-option items Q2–Q5) to 5 (six-option items on DS2); the item-weighted maximum across the 32 information items is approximately 2.9. Multi-select answers (DS2 Q6/Q7 and Q9 across all DS) are split into per-option votes when computing distributions and modes.

|  | | **Modal MAE** | | | **EAD** | | |
| --- | --- | --- | --- | --- | --- | --- | --- |
| **Type** | **DS** | **Edu-Low** | **Edu-High** | **Δ** | **Edu-Low** | **Edu-High** | **Δ** |
| Perception | DS1: Short + Difficult | 1.50 | 0.00 | **+1.50** | 1.20 | 0.86 | +0.35 |
|  | DS2: Long + Simple | 1.50 | 0.50 | +1.00 | 1.17 | 0.79 | +0.38 |
|  | DS3: Long + Difficult | 1.50 | 1.00 | +0.50 | 1.32 | 0.77 | **+0.55** |
|  | DS4: Short + Simple | 2.00 | 1.50 | +0.50 | 1.48 | 1.00 | +0.48 |
|  | Overall | 1.63 | 0.75 | +0.88 | 1.29 | 0.85 | +0.44 |
| Information | DS1: Short + Difficult | 0.25 | 0.38 | −0.13 | 0.44 | 0.52 | −0.07 |
|  | DS2: Long + Simple | 1.50 | 1.25 | +0.25 | 1.21 | 1.16 | +0.05 |
|  | DS3: Long + Difficult | 1.13 | 0.88 | +0.25 | 0.98 | 1.02 | −0.03 |
|  | DS4: Short + Simple | 0.75 | 1.00 | −0.25 | 1.00 | 1.01 | −0.01 |
|  | Overall | 0.91 | 0.88 | +0.03 | 0.91 | 0.93 | −0.02 |

## 4.5 Gender Gap by Discharge-Summary Type

To assess whether gender-stratified alignment showed a discharge-summary-dependent pattern comparable to the education gap, we computed per-discharge-summary gender gaps, defined as male minus female alignment distance, for all four models on perception-item modal MAE (Table S7). Unlike the education gap, the gender gap did not exhibit a consistent document-level pattern across models. No model showed a unidirectional gender gap across all four discharge summaries; each model had at least one summary for which the gap was zero or reversed direction. By contrast, Sonnet 4.5 showed a positive education gap, indicating poorer low-education alignment, on all four summaries (Table S7). This directional inconsistency suggests that gender-stratified alignment differences were driven primarily by question- or document-specific modal shifts rather than by a stable, systematic gender-based alignment gap.

Isolated but large gender gaps did occur for some models on specific summaries. GPT-5.2 showed a gender gap of +1*.*50 on DS3, the long-and-difficult summary, which was the largest gender-related cell-level disparity in the analysis. GPT-4.1 showed gaps of +1*.*00 on both DS3 and DS4. These peaks were offset, however, by zero or reversed gaps on other summaries, yielding more moderate overall perception-item gaps of +0*.*38 for GPT-5.2 and +0*.*62 for GPT-4.1.

The corresponding EAD gender gaps were small across all models and discharge summaries (|∆| ≤ 0*.*28), indicating that the modal MAE differences largely reflected sensitivity to modal-response switching rather than broad distributional mismatch. Taken together, these results suggest that discharge-summary characteristics more systematically influenced education-stratified alignment than gender-stratified alignment. In this analysis, education level emerged as the more consistent equity concern for LLM-based persona simulation in evaluations of patient-centered communication.

Table S7: Per-discharge-summary gender gap on perception modal MAE (male minus female) for all four models. Positive values indicate poorer alignment for the male subgroup; bold values indicate the largest absolute gap per model.

| **DS Type** | **Sonnet 4.5** | **Opus 4.6** | **GPT-5.2** | **GPT-4.1** |
| --- | --- | --- | --- | --- |
| DS1: Short & Difficult | +0.50 | 0.00 | +0.50 | +0.50 |
| DS2: Long & Simple | 0.00 | 0.00 | 0.00 | 0.00 |
| DS3: Long & Difficult | +0.50 | −0.50 | **+1.50** | **+1.00** |
| DS4: Short & Simple | −0.50 | **+0.50** | −0.50 | **+1.00** |
| Overall | +0.13 | 0.00 | +0.38 | +0.63 |

## 4.6 Misalignment Analysis Details

Table S8: Per-question misalignment summary for Sonnet 4.5 on information-based questions (*n* = 32). The modal distance is the ordinal distance between the LLM modal response and the human modal response. The per-item maximum attainable distance varies across the 32 items (2 for three-option items Q2–Q5; 3 for four-option items; 4 for five-option items; 5 for six-option items on DS2); the "2+" bucket therefore captures all items whose modal distance reaches half of their respective maxima or more. Multi-select answers are split into per-option votes when computing the human modal response.

| **Modal Distance** | **Count** | **%** |
| --- | --- | --- |
| 0 (perfect match) | 11 | 34.4 |
| 1 (off by one) | 14 | 43.8 |
| 2+ (off by two or more) | 7 | 21.9 |

Table S9: Seven worst-aligned information questions for Sonnet 4.5 (modal distance ≥ 2). Response distributions are shown as the percentage of each option chosen. Human *n* ≈ 75 per question; LLM *n* ≈ 7,680 pooled across 10 iterations and all personas. The per-DS topic of each item is reported, since Q-numbers reuse across discharge summaries even though the underlying clinical content varies (notably Q6 on DS2 = "when to call the doctor," Q7 = "activities prohibited" on DS2 and an orthopedic variant on DS3, and Q9 across all DS = multi-select discharge-plan coverage). For multi-select items (DS2 Q6/Q7 and Q9 across DS), each chosen option contributes one vote to the response distribution; trace levels (< 1%) are omitted from the displayed cells.

| **Question** | **Topic** | **Hum** | **LLM** | **Dist** | **Human Distribution** | **LLM Distribution** |
| --- | --- | --- | --- | --- | --- | --- |
| DS2-Q6 | When to call doctor | A | E | 4 | A:34% B:33% D:32%* | E:100% |
| DS1-Q9 | Discharge plan coverage | D | B | 2 | A:19% C:22% D:22% E:20% | B:71% D:29% |
| DS2-Q7 | Activities prohibited | B | D | 2 | B:41% C:21% D:37% | B:40% C:11% D:48% |
| DS2-Q9 | Discharge plan coverage | A | C | 2 | A:30% B:22% D:16% E:11% G:8% | C:40% D:37% E:17% B:6% |
| DS3-Q5 | Other prescriptions identified | C | A | 2 | A:40% B:10% C:50% | A:100% |
| DS3-Q9 | Discharge plan coverage | C | E | 2 | A:20% B:14% C:24% E:21% | E:61% C:39% |
| DS4-Q5 | Other prescriptions identified | C | A | 2 | A:11% B:9% C:80% | A:100% |

* E < 1%

## 4.7 Exact-match and Per-item Normalized Information-question Alignment

Table S10: Exact-match alignment of LLM modal responses to the pooled human modal response, reported for all 32 information-based questions and split by question type. Q2–Q5 form the confidence/certainty axis (3-option “Yes” / “I don’t know” / “Not provided”), and Q6–Q9 are categorical and presentation-ordered. Top-1 modal match is the proportion of items where the LLM modal response equals the human modal response; mean P (LLM = human mode) is the mean across items of the LLM response probability on the human-modal option. Opus 4.6 achieves the highest exact-match alignment on the information-based items, and Sonnet 4.5 the lowest.

| **Model** | **All Q2–Q9 (n=32) Top-1 / Mean P** | **Q2–Q5 confidence (n=16) Top-1 / Mean P** | **Q6–Q9 categorical (n=16) Top-1 / Mean P** |
| --- | --- | --- | --- |
| Opus 4.6 | 62.5% / 57.9% | 93.8% / 82.5% | 31.2% / 33.4% |
| GPT-5.2 | 46.9% / 50.5% | 62.5% / 64.6% | 31.2% / 36.3% |
| GPT-4.1 | 50.0% / 44.5% | 62.5% / 54.3% | 37.5% / 34.6% |
| Sonnet 4.5 | 34.4% / 34.6% | 25.0% / 24.3% | 43.8% / 45.0% |

Table S11: Per-item normalized information-question alignment. For each of the 32 information items, the modal mean absolute error and expected absolute distance are divided by that item’s own theoretical maximum (2 for three-option items Q2–Q5; 3 for four-option items Q7 on DS1/DS4 and Q8 across all DS; 4 for five-option items Q6 on DS1/DS3/DS4, Q7 on DS3, and Q9 across all DS; 5 for six-option items Q6 and Q7 on DS2) before averaging across items. All values therefore lie on a uniform [0, 1] scale where lower indicates closer alignment with the matched human subgroup. Opus 4.6 achieves the lowest normalized modal MAE and EAD on the information-question block, and Sonnet 4.5 the highest.

| **Normalized Modal MAE** | **Education** | | **Gender** | | | **Doctor Visits** | | **ER Visits** |
| --- | --- | --- | --- | --- | --- | --- | --- | --- |
|  | Low | High | Male | Female | | Low | High | Low |
| Sonnet 4.5 | 0.35 | 0.33 | 0.32 | 0.33 | 0.34 | | 0.35 | 0.33 |
| Opus 4.6 | 0.22 | 0.18 | 0.21 | 0.18 | 0.24 | | 0.18 | 0.18 |
| GPT-5.2 | 0.27 | 0.18 | 0.23 | 0.26 | 0.26 | | 0.24 | 0.21 |
| GPT-4.1 | 0.32 | 0.27 | 0.25 | 0.25 | 0.25 | | 0.24 | 0.25 |
| **Normalized Modal EAD** | | | | |  | |  |  |
| Sonnet 4.5 | 0.34 | 0.34 | 0.34 | 0.35 | 0.35 | | 0.34 | 0.34 |
| Opus 4.6 | 0.26 | 0.28 | 0.28 | 0.26 | 0.27 | | 0.27 | 0.27 |
| GPT-5.2 | 0.31 | 0.28 | 0.30 | 0.29 | 0.30 | | 0.30 | 0.29 |
| GPT-4.1 | 0.33 | 0.32 | 0.33 | 0.33 | 0.32 | | 0.34 | 0.33 |

1. In the experiment, we replaced the stakeholder with one parameter from each category. We evaluated all possible parameter combinations and repeated each combination 10 times for LLM queries. However, due to the limited racial diversity among human participants, we did not consider the influence of race, although race-related information was included in the prompts.
2. * We insert an additional question: “Select 4 if you see this question: A. 1 B. 2 C. 3 D. 4,” at the end of the questionnaire as the attention check question.
